# Multifunctional Superparticles for Magnetically Targeted NIR‐II Imaging and Photodynamic Therapy

**DOI:** 10.1002/advs.202203669

**Published:** 2022-11-22

**Authors:** Yilin Liu, Yuan Liang, Pengpeng Lei, Zhen Zhang, Yongming Chen

**Affiliations:** ^1^ School of Materials Science and Engineering Sun Yat‐sen University Guangzhou 510275 P. R. China; ^2^ State Key Laboratory of Rare Earth Resource Utilization Changchun Institute of Applied Chemistry Chinese Academy of Sciences 5625 Renmin Street Changchun 130022 P. R. China; ^3^ School of Rare Earths University of Science and Technology of China Hefei 230026 P. R. China; ^4^ Ganjiang Innovation Academy Chinese Academy of Sciences Ganzhou Jiangxi 341000 P. R. China

**Keywords:** magnetic targeting, multifunction, NIR‐II imaging, photodynamic therapy, upconversion

## Abstract

Theranostics, the combination of diagnostics and therapies, has been considered as a promising strategy for clinical cancer treatment. Nonetheless, building a smart theranostic system with multifunction for different on‐demand applications still remains elusive. Herein, an easy and user‐friendly microemulsion based method is developed to modularly assemble upconversion nanoparticles (UCNPs) and Fe_3_O_4_ nanoparticles together, forming multifunctional UCNPs/Fe_3_O_4_ superparticles with highly integrated functionalities including the 808 nm excitation for real‐time NIR‐II imaging, magnetic targeting, and the upconversion luminescence upon 980 nm excitation for on‐demand photodynamic therapy (PDT). With a magnet placed nearby the tumor, in vivo NIR‐II imaging uncovers that superparticles tend to migrate toward the tumor and exhibit intense tumor accumulation, ≈6 folds higher than that without magnetic targeting 2 h after intravenous injection. NIR laser irradiation is then used to trigger PDT, obtaining an outstanding tumor elimination under magnetic tumor targeting, which shows a high potential to be applied in targeted cancer theranostics.

## Graphical abstract

An easy and user‐friendly microemulsion based method is developed to modularly assemble upconversion nanoparticles (UCNPs) and Fe_3_O_4_ nanoparticles together, forming a multifunctional UCNPs/Fe_3_O_4_ superparticles with highly integrated functionalities including real‐time NIR‐II imaging, magnetic targeting, and photodynamic therapy (PDT), which shows a high potential to be applied in cancer theranostics.

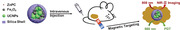

## 1 Introduction

In the past decades, multifunctional nanoplatforms have achieved great progress in a broad range of applications, because they could integrate multicomponent functional materials into a single entity through rational designing of hierarchical structures. Among various stimuli utilized in multifunctional nanoplatforms, such as temperature, pH, enzymes, and reduction/oxidation, light is particularly favored owing to its ease of activation and high spatial and temporal control. Light‐based multifunctional nanoplatforms have been used in biomedical fields, however, the majority of light‐sensitive molecules are only sensitive to UV or visible light, which suffer from limitations, such as phototoxicity (UV) and low tissue penetration capabilities (UV–visible).^[^ ^1^, ^2^, ^3^, ^4^, ^5^, ^6^, ^7^, ^8^ ^]^ Upconversion nanoparticles (UCNPs) doped with lanthanides are one of the most popular used light‐responsive nanomaterials as they act as a nanotransducer to upconvert NIR light to UV–visible light with a high tissue penetration depth and low phototoxicity in biological tissues.^[^ ^9^, ^10^, ^11^, ^12^, ^13^, ^14^, ^15^, ^16^, ^17^, ^18^, ^19^, ^20^, ^21^, ^22^ ^]^ Over the past decades, many researchers have applied UCNPs in biomedical applications, particularly NIR‐light induced photodynamic therapy (PDT) in vitro and in vivo.^[^ ^23^, ^24^, ^25^, ^26^, ^27^, ^28^ ^]^ Except for classical cancer therapies such as surgery, radiotherapy, and chemotherapy, PDT has recently emerged as an alternative and promising treatment for the control of malignant diseases.^[^ ^29^, ^30^, ^31^ ^]^ However, PDT meant for practical cancer treatment calls for not only real‐time imaging to track the distribution of nanoparticles in tumors but also tumor targeting ability to ensure high treatment efficacy and reduce side effects.^[^ ^32^, ^33^, ^34^, ^35^, ^36^, ^37^, ^38^, ^39^ ^]^ Compared to conventional molecular modification‐based tumor targeting, physical interaction‐based magnetic targeting employs a magnetic field to attract magnetic nanoparticles that circulate in the blood to the tumor site where the magnet is placed, which avoids the specific receptor expression restriction and complex targeting molecule modification, indicating to be a more universal approach for tumor targeting.^[^ ^40^, ^41^, ^42^, ^43^ ^]^ To establish a PDT platform with effective tumor‐targeting efficacy, a composite consisting of UCNPs and Fe_3_O_4_ has great potential.

To date, three strategies have been mainly designed for the fabrication of UCNPs/Fe_3_O_4_ composites and their utilization in various applications. One is the mesoporous silica (SiO_2_)‐loading UCNPs or Fe_3_O_4_ nanoparticles with ultrasmall size (below 10 nm) inside the mesoporous.^[^ ^44^, ^45^, ^46^ ^]^ The second is the cross‐linker anchoring method.^[^ ^47^, ^48^, ^49^ ^]^ Still, this process requires time‐consuming and complex surface modification before bonding two nanoparticles together, and it is hard to control the bonding process as one nanoparticle could act as a linker to hold the other nanoparticle aggregate together to form macroaggregates or even precipitates. The third is the seed‐induced growth method to synthesize monodisperse core–shell nanocomposites.^[^ ^37^, ^50^, ^51^, ^52^ ^]^ The Fe_3_O_4_ nanoparticles play the role of the core which enables the nanocrystals with magnetic property, while the UCNPs outer shell induces the upconversion property, which involves troublesome epitaxial growth of shell layers. In short, it still remains a great challenge to efficiently combine these two nanoparticles in a simple and controllable manner.

To overcome these problems, here a simple yet versatile microemulsion based method was designed to coassemble UCNPs and Fe_3_O_4_ nanoparticles together, forming multifunctional superparticles (MFSPs). This only requires the synthesis of individual (UCNPs and Fe_3_O_4_) nanoparticles separately and then assembles two nanoparticles to form an entity (superparticles) and various parameters such as the number of individual nanoparticles, their size/shape and the size of the superparticles can be precisely controlled. Compared with previous complicated synthesis for UCNPs/Fe_3_O_4_ composites, this method is easier, faster, and more versatile. Mesoporous silica shell was further deposited onto the MFSPs and photosensitizer then anchored to mesoporous silica to obtain a magnetically targeted real‐time NIR‐II imaging and PDT, in which Fe_3_O_4_ nanoparticles within MFSPs could help MFSPs accumulate around tumors by placing a magnet nearby the tumor, NIR‐II emissions upon 808 nm laser irradiation was utilized for the real‐time imaging and the green emissions upon 980 nm laser excitation was to generate reactive oxygen species (ROS) for efficiently on‐demand PDT. Compared with other reported NIR‐II imaging‐guided PDT systems in which the NIR imaging and PDT are difficult to control separately, and photoactivation for imaging leads to simultaneous PDT that results in undesired cell killing,^[^ ^53^, ^54^, ^55^, ^56^, ^57^, ^58^, ^59^, ^60^ ^]^ the developed superparticles with orthogonal activation capability here were employed to achieve this in a controlled manner: 808 nm irradiation helped in NIR‐II imaging, and 980 nm irradiation was used to generate ROS for PDT. Only these two events are performed in a subsequent manner, real bioimaging and controlled cell killing could be ensured. This suggests that these MFSPs could offer great potential for NIR light triggered and targeted nanoplatforms for real‐time imaging guided therapy.

## 2 Synthesis and Characterization of MFSPs

**Figure 1:**
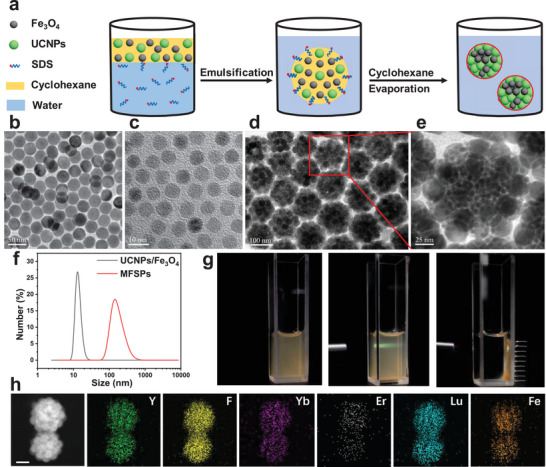
a) A scheme showing the micro‐emulsion based synthesis method for the coassembly of UCNPs and Fe_3_O_4_ nanoparticles into UCNPs/Fe_3_O_4_ MFSPs. TEM images of UCNPs b), Fe_3_O_4_ nanoparticles c), MFSPs d), and magnified figure of MFSPs e). f) DLS results of mixtures of UCNPs and Fe_3_O_4_ in cyclohexane (weight ratio is 1:1) and UCNPs/Fe_3_O_4_ MFSPs in water. g) Photos of UCNPs/Fe_3_O_4_ MFSPs in water under ambient light (left), 980 nm laser irradiation (middle), and magnetic field (right). h) High‐angle annular dark‐field scanning transmission electron microscopy (HADDF‐STEM) image, and STEM elemental mapping of Y, F, Yb, Er, Lu, and Fe. UCNPs: NaYF_4_: 20% Yb, 2% Er@ NaLuF_4_: 25%Y.

The UCNPs and Fe_3_O_4_ nanoparticles were assembled into multifunctional superparticles (MFSPs) via a microemulsion‐based synthesis method^[^ ^61^, ^62^, ^63^ ^]^ as illustrated in **Figure** **1**a. Initially, a commonly used green emitting core–shell UCNPs (NaYF_4_: 20%Yb, 2%Er@NaLuF_4_: 25%Y) with a diameter of 35 nm^[^ ^64^ ^]^ and Fe_3_O_4_ nanoparticle with a diameter of 8 nm^[^ ^65^ ^]^ (Figure 1b,c) were selected to demonstrate the effectiveness of this approach to synthesize UCNPs/Fe_3_O_4_ superparticles. The oil phase (cyclohexane, 1 mL) containing well‐dispersed oleic acid (OA)‐capped UCNPs and Fe_3_O_4_ nanoparticles (5 mg mL^−1^, the weight ratio is 1:1) were mixed with a 10 mL aqueous phase containing anionic surfactant (sodium dodecyl sulfate (SDS), 0.6 mg mL^−1^) under sonication and vigorous stirring to obtain a stable oil/water emulsion mixture (Figure S1, Supporting Information). The well‐dispersed UCNPs and Fe_3_O_4_ nanoparticles were confined within emulsion droplets which are stabilized by the SDS. Subsequently, during the evaporation process of low‐boiling cyclohexane at 70 °C for 4 h, the emulsion droplets shrunk accordingly, inside which the two nanoparticles got concentrated, aggregated, and packed closely with each other, thereby assembling to UCNPs/Fe_3_O_4_ MFSPs as schematically illustrated in Figure 1a. Through hydrophobic Van der Waals interactions, the alkane chains of SDS from the aqueous phase interdigitated spontaneously with the OA's alkane chains located outside the surface of MFSPs to ensure the resultant spherical MFSPs can be well dispersed in an aqueous solution.^[^ ^66^ ^]^ The presence of SDS on the surfaces of MFSPs was confirmed by a negative surface zeta potential (about −25 mV, Table S1, Supporting Information). The averaged hydrodynamic diameter of MFSPs evaluated by dynamic light scattering (DLS) was around 170 nm (Figure 1f, red line) which was significantly larger than that of the mixtures of UCNPs and Fe_3_O_4_ nanoparticles in cyclohexane (about 14 nm, Figure 1f, black line). In addition, as the TEM illustrated in Figure 1d, the UCNPs and Fe_3_O_4_ nanoparticles coassembled to form spherical MFSPs, and the UCNPs and Fe_3_O_4_ nanoparticles did not sinter into larger units as two different sized spherical nanoparticles consistent with UCNPs and Fe_3_O_4_ nanoparticles existed within one MFSP (Figure 1e). The prepared MFSPs consisting of two functional nanoparticles exhibited their effective performances, respectively: green‐colored upconversion luminescence excited by a 980 nm laser and directional movement under the control of a magnet (Figure 1g). All MFSPs showing both functions at the same time confirmed that UCNPs and Fe_3_O_4_ nanoparticles are combined within one single MFSP, which was further proven by the elemental mapping of Y, F, Yb, Er, Lu, Fe, Na, and O in scanning transmission electron microscopy (STEM), indicating the colocalization of lanthanide elements and Fe element with uniform distribution within MFSPs (Figure 1h; and Figure S2, Supporting Information).

## 3 Construction of MFSPs@mSiO_2_‐ZnPC Nanophotosensitizers

**Figure 2:**
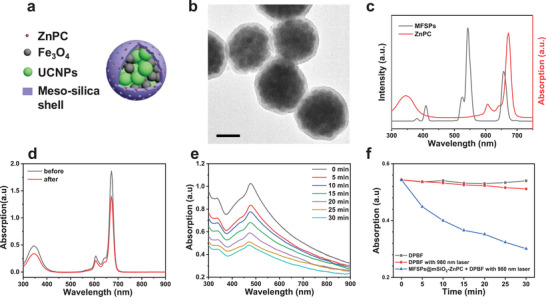
a) Schematic illustrations of MFSPs@mSiO_2_‐ZnPC. b) TEM images of MFSPs@mSiO_2_. c) The emission spectrum of MFSPs under 980 nm laser irradiation (black line) and UV–vis absorption spectrum of ZnPc (red line). d) The UV–vis absorption of ZnPC in DMSO solution (black line) and its supernatant after loading ZnPC into MFSPs@mSiO_2_. e) UV–vis absorption spectra of MFSPs@mSiO_2_‐ZnPC with DPBF indicators after 980 nm laser irradiation for different time. f) The absorption quenching comparison of DPBF for three different groups: (1) DPBF in DMSO solution, (2) DPBF in DMSO solution under 980 nm laser irradiation (0.8 W cm^−2^), and (3) DPBF with MFSPs@mSiO_2_‐ZnPC in aqueous solution under 980 nm laser irradiation (0.8 W cm^−2^).

For potential biomedical applications, such as cancer theranostics, a biocompatible mesoporous silica shell was subsequently uniformly coated on the MFSPs (MFSPs@mSiO_2_) as shown in **Figure** **2**a; and Figure S3 (Supporting Information). Figure 2b demonstrated the size and morphology of MFSPs@mSiO_2_ with a thickness of about 18 nm, which indicated that the MFSPs were well encapsulated by a uniform mesoporous silica shell which allowed for a great number of molecule storage due to their high surface area and mesoporous structure.^[^ ^67^, ^68^, ^69^, ^70^, ^71^ ^]^ In addition, the MFSPs@mSiO_2_ were tested for their stability. It was seen that the MFSPs@mSiO_2_ were highly stable in deionized water, pH = 4 water solution, and in 10% fetal bovine serum solution over a period of 72 h, as shown in Figure S4 (Supporting Information). Then, to achieve an effective PDT efficacy, the MFSPs@mSiO_2_ were further loaded with photosensitizers, specifically, zinc phthalocyanine (ZnPC), as their strong absorption (600–700 nm) overlapped well with the green emission band from the UCNPs within MFSPs@mSiO_2_ under 980 nm laser irradiation. The loading quantity of ZnPC in MFSPs@mSiO_2_ was found to be 2.67 wt% by UV–vis spectrophotometry measurement (Figure 2d). Additionally, the release of ZnPC from MFSPs@mSiO_2_‐ZnPC was studied by soaking the MFSPs@mSiO_2_‐ZnPC in dimethyl sulfoxide (DMSO), deionized water, PBS buffer, and cell culture medium (DMEM) for 24 h, respectively. Through the UV–vis absorption characterization on collected supernatants, the hydrophobic ZnPC molecules were found to be encapsulated well inside mesoporous silica when in an aqueous solution (Figure S5, Supporting Information). The metabolic pathway and biodistribution of the MFSPs@mSiO_2_‐ZnPC in mice were determined by quantifying the concentration and % injected dose of Fe in various major organs: liver, spleen, kidneys, lungs, and heart, at different time points from 0.5 h to 14 days postinjection using inductively coupled plasma mass spectrometry (ICP‐MS), as shown in Figure S6 (Supporting Information). Fe is a component of MFSPs@mSiO_2_‐ZnPC and its concentration in biological tissues is directly proportional to the concentration of MFSPs@mSiO_2_‐ZnPC. MFSPs@mSiO_2_‐ZnPC could be detected primarily with a high percentage in the liver and spleen, with a much lower degree of accumulation detected in the heart, lung, and kidney. After 8 h a decrease in accumulation of Fe in all organs (especially the liver and spleen) was observed, particularly when comparing between 8 h and 14 days postinjection, indicating the potential clearance of the MFSPs@mSiO_2_‐ZnPC from the liver and spleen. With the liver and spleen being major organs of the mononuclear phagocytes system (MPS), the MFSPs@mSiO_2_‐ZnPC accumulation observed in these two organs was likely a result of clearance by Kupffer cells in the liver and spleen macrophages. Following intravenous injection of MFSPs@mSiO_2_‐ZnPC, these particles were opsonized and cleared from the blood circulation over time by these macrophages via phagocytosis.

The ROS generation from the MFSPs@mSiO_2_‐ZnPC under 980 nm laser irradiation was evaluated by using a fluorescence dye, the 1,3‐diphenylisobenzofuran (DPBF), which has a highly specific quenching manner toward ROS, leading to an indicative absorption intensity decrease.^[^ ^72^ ^]^ As shown in Figure 2e upon the irradiation with 980 nm laser irradiation, the absorbance of DPBF at 417 nm decreased rapidly in the presence of MFSPs@mSiO_2_‐ZnPC, indicating that ROS were generated significantly from MFSPs@mSiO_2_‐ZnPC upon 980 nm excitation. Furthermore, Figure 2f indicated that the absorbance of DPBF showed no obvious variation in the absence of MFSPs@mSiO_2_‐ZnPC no matter whether the 980 nm laser irradiation existed or not, demonstrating that the ROS was generated from MFSPs@mSiO_2_‐ZnPC.

## 4 Imaging‐Guided PDT for Cancer Therapeutics

**Figure 3:**
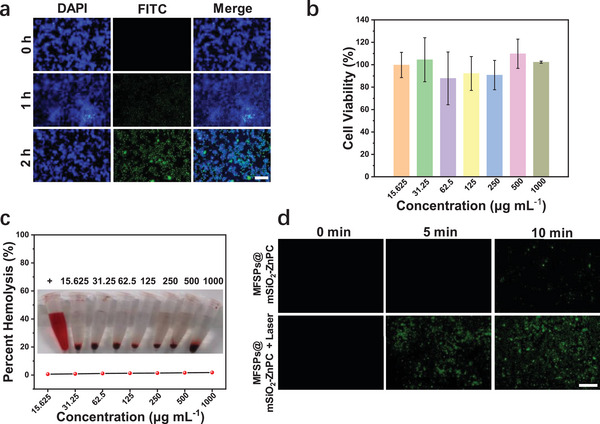
a) Cellular uptake of MFSPs@mSiO_2_‐ZnPC visualized by fluorescence microscopy. b) In vitro cytotoxicity of 4T1 cells after incubation with MFSPs@mSiO_2_‐ZnPC of different concentrations for 24 h. c) Percentage of erythrocytes hemolysis incubated with different concentrations of MFSPs@mSiO_2_‐ZnPC for 4 h. d) Detection of the ROS generation from MFSPs@mSiO_2_‐ZnPC in vitro under different 980 nm laser irradiation (0.8 W cm^−2^) for diverse incubation time (0, 5, and 10 min). Scale bar = 100 µm.

Before applying the MFSPs@mSiO_2_‐ZnPC for PDT, the cellular uptake and cytotoxicity were evaluated necessarily. Cellular uptake and distribution of MFSPs@mSiO_2_‐ZnPC in 4T1 cells were first visualized under inverted fluorescence microscopy, in which, the visualization of the cell nucleus was achieved by DAPI (blue), while the green luminescence originated from the FITC‐labeled MFSPs@mSiO_2_‐ZnPC (Table S1 and Figure S7, Supporting Information). Besides, by merging the DAPI and FITC signals in the figures, the distribution of MFSPs@mSiO_2_‐ZnPC was mainly aggregated around the nucleus, certifying the effective cellular uptake of MFSPs@mSiO_2_‐ZnPC (**Figure** **3**a). Figure 3b presented the cytotoxicity of 4T1 cells incubated with different concentrations (15.625, 31.25, 62.5, 125, 250, 500, and 1000 µg mL^−1^) of MFSPs@mSiO_2_‐ZnPC. The cell viability was still over 90% even the concentration was up to 1000 µg mL^−1^, suggesting that MFSPs@mSiO_2_‐ZnPC showed negligible cytotoxicity to 4T1 cells in concentration ranges up to 1000 µg mL^−1^. Hemolysis represented the damage to red blood cells (RBCs) and resulted in the release of the iron‐containing protein hemoglobin into plasma, thereby rejecting nanomaterials in vivo experiments. Figure 3c showed that MFSPs@mSiO_2_‐ZnPC hardly induced hemolysis over a wide range of concentrations of MFSPs@mSiO_2_‐ZnPC, indicating a safe performance for in vivo experiments. In addition, in order to visualize the effective ROS production from MFSPs@mSiO_2_‐ZnPC inside cancer cells, the 2’,7’‐dichlorofluorescein‐diacetate (DCFH‐DA) probe was employed to characterize the ROS production because the oxidation of DCFH by ROS could yield a strong green fluorescence signal.^[^ ^73^ ^]^ Figure 3d demonstrated the gradual generation of ROS inside cells from MFSPs@mSiO_2_‐ZnPC with the 980 nm laser irradiation.

## 5 PDT Effects In Vitro

**Figure 4:**
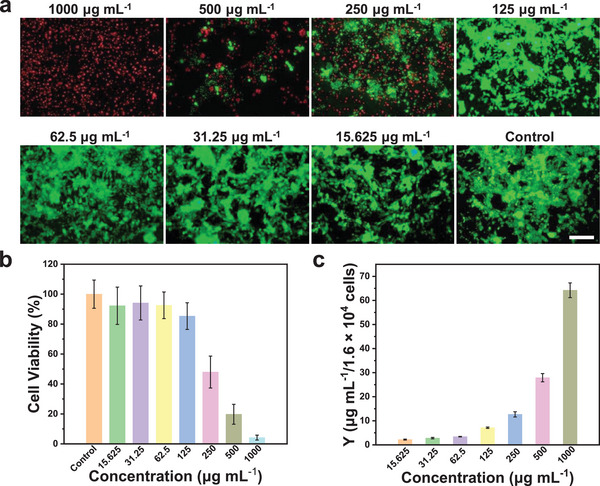
a) The live/dead assay of 4T1 cells after treatment with different concentrations of MFSPs@mSiO_2_‐ZnPC. Live cells and dead cells were stained respectively by Calcein‐AM (green) and PI (red). Scale bar = 100 µm. b) Detection of in vitro viability of 4T1 cells under 980 nm laser irradiation (0.8 W cm^−2^) for 10 min with different concentrations of MFSPs@mSiO_2_‐ZnPC. c) Analysis of cellular uptake capacity for different concentrations of MFSPs@mSiO_2_‐ZnPC by ICP‐MS.

To evaluate the PDT effects of MFSPs@mSiO_2_‐ZnPC, the 4T1 cells were incubated with MFSPs@mSiO_2_‐ZnPC at different concentrations under 980 nm laser irradiation. To demonstrate the cell killing capacity intuitively, the live/dead cell viability staining was performed in **Figure** **4**a, which indicated distinct emergence and enhancement of dead cells (red color) with the increased MFSPs@mSiO_2_‐ZnPC concentration in each figure under 980 nm laser irradiation. When incubated with a high concentration up to 1000 µg mL^−1^ of MFSPs@mSiO_2_‐ZnPC, the cell viability was only 4% even under low irradiation power (0.8 W cm^−2^) for 10 min, indicating an effective cell killing efficacy of MFSPs@mSiO_2_‐ZnPC, as summarized in Figure 4b. This was also consistent with the cellular uptake results in Figure 4c that cellular uptake capacity is higher when incubated with more MFSPs@mSiO_2_‐ZnPC as evidenced by the quantification of the Y element in cells through the ICP‐MS test, which then could generate more ROS to cause cell death under same power intensity of 980 nm laser irradiation.

## 6 Magnetic Targeting and Real‐Time Bioimaging In Vivo

**Figure 5:**
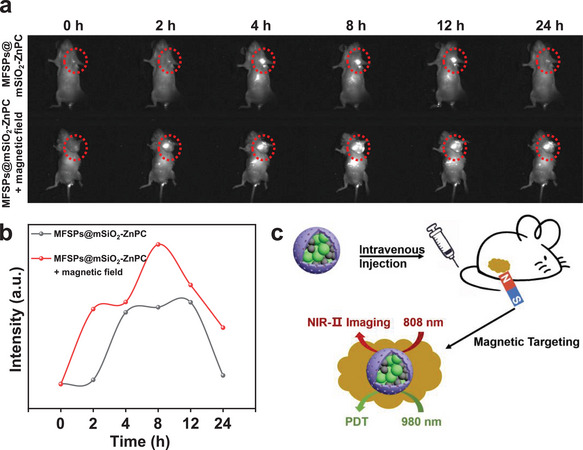
a) In vivo NIR‐II bioimaging of mice under 808 nm laser irradiation (0.2 W cm^−2^ power density, 100 ms). b) Fluorescence intensity versus time at tumor sites in mice. c) Schematic illustration of magnetically targeted NIR‐II bioimaging and PDT in mice.

The magnetic targeting performance of MFSPs@mSiO_2_‐ZnPC was carried out in balb/c female mice bearing 4T1 murine breast cancer tumors by intravenously injecting MFSPs (100 µL, 1000 µg mL^−1^) with a small magnet placed nearby the tumor. Furthermore, the real‐time NIR‐II bioimaging of MFSPs@mSiO_2_‐ZnPC was achieved by the strong downshifting emission at the range of about 1000–1600 nm of aggregated ZnPC inside mesoporous pores with the 808 nm laser excitation (Figure S8, Supporting Information).^[^ ^74^, ^75^, ^76^ ^]^ Moreover, as indicated in Figure S9 (Supporting Information), very little ROS was produced by MFSPs@mSiO_2_‐ZnPC upon 808 nm laser irradiation, therefore the 808 nm laser irradiated NIR‐II emission from ZnPC could be employed for real‐time bioimaging and 980 nm triggered PDT monitoring. Specifically, after intravenous injection of MFSPs@mSiO_2_‐ZnPC into mice, in the presence of an external magnet tied to the skin area near the tumor, the NIR‐II signal under 808 nm laser irradiation was recorded over time to track the MFSPs@mSiO_2_‐ZnPC distribution. **Figure** **5**a indicated that the ultrabright emission was observed in the tumor regions, suggesting the high accumulation of MFSPs@mSiO_2_‐ZnPC toward the tumor with the help of an external magnetic field. Mice injected with MFSPs@mSiO_2_‐ZnPC in the absence of the external magnetic field as the control group exhibited a rather lower accumulation of MFSPs@mSiO_2_‐ZnPC in the tumor regions. Quantification of NIR‐II fluorescence intensity presented about 6 folds higher fluorescence signals in the tumor regions in the presence of the magnetic field 2 h after injection, and the magnetic field also helped the MFSPs@mSiO_2_‐ZnPC reach a higher enrichment level in a shorter time (Figure 5b). After a longer time postinjection, up to 24 h, the NIR‐II fluorescence signal still exhibited an excellent tumor accumulation, rather than cleared by blood rapidly, indicating the superior magnetic targeting efficiency and long residence of MFSPs@mSiO_2_‐ZnPC toward tumors, which had a great potential in higher and longer PDT potency (Figure 5c).

## 7 Magnetically Targeted PDT

**Figure 6:**
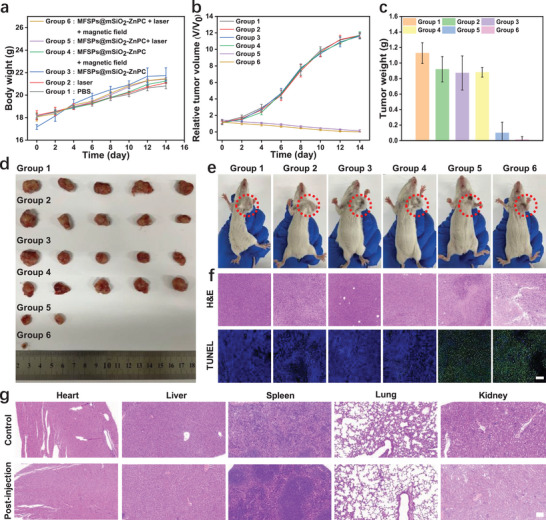
In vivo PDT efficacy of MFSPs@mSiO_2_‐ZnPC. Group 1–6 are PBS, only 980 nm laser irradiation, MFSPs@mSiO_2_‐ZnPC, MFSPs@mSiO_2_‐ZnPC + magnetic field, MFSPs@mSiO_2_‐ZnPC + 980 nm laser irradiation, and MFSPs@mSiO_2_‐ZnPC + magnetic field + 980 nm laser irradiation, respectively. Body weight a) and relative tumor volume b) and tumor weight c) in different treatment groups. The photographs of excised tumors d) and tumor‐bearing mice e) in different groups at the end of treatment. f) Representative tumor sections stained with H&E and TUNEL assay of corresponding mice in e). g) Histological comparison in the heart, liver, spleen, lung, and kidney between normal mice and the mice 28 days after intravenous injection of MFSPs@mSiO_2_‐ZnPC (1000 µg mL^−1^, 100 µL). Scale bars = 100 µm.

More importantly, magnetically targeted PDT toward tumors was performed subsequently by injecting 100 µL of PBS or 100 µL MFSPs@mSiO_2_‐ZnPC (1000 µg mL^−1^) dissolved in PBS into each tumor‐bearing mouse. Six groups of mice were treated as follows: the Group 1, 2, 3, 4, and 5 were treated with PBS, only 980 nm laser irradiation, MFSPs@mSiO_2_‐ZnPC in PBS solution, MFSPs@mSiO_2_‐ZnPC in PBS solution with magnetic field, and MFSPs@mSiO_2_‐ZnPC in PBS solution and later irradiated with 980 nm, respectively, while the Group 6 was injected with MFSPs@mSiO_2_‐ZnPC in PBS solution with magnetic field and later irradiated with 980 nm laser. External magnets tied near the tumor regions were placed on mice to assist with the MFSPs@mSiO_2_‐ZnPC accumulation in tumor regions in vivo treatment experiments. To ensure a fair comparison, the body weight of each mouse was measured every 2 days after the initial treatment and all groups suggested a slow but increased tendency (**Figure** **6**a). 8 h after postintravenous injection, the mice were anesthetized and the tumor sites were irradiated with/without 980 nm laser irradiation accordingly (0.8 W cm^−2^, 10 min). Afterward, the tumor volumes of each group were measured to characterize the PDT efficacy. As seen in Figure 6b,c, mice in the Group 1 (PBS), Group 2 (only 980 nm laser irradiation), Group 3 (MFSPs@mSiO_2_‐ZnPC), and Group 4 (MFSPs@mSiO_2_‐ZnPC + magnetic field) exhibited significant tumor volume increase, while the Group 5 (MFSPs@mSiO_2_‐ZnPC + 980 nm laser irradiation) shown the tumor growth was inhibited in comparison to the Group 1–4 (Figure 6b–e), and the Group 6 with magnetic field shown the best PDT efficacy, demonstrating the advantage of magnetic targeting to assist with the MFSPs@mSiO_2_‐ZnPC accumulation in tumor regions. In addition, tumors excised from corresponding mice were compared intuitively, showing that only mice treated with both MFSPs@mSiO_2_‐ZnPC and 980 nm laser irradiation exhibited the smallest tumor size (Figure 6c–e). To further explore the mechanisms of PDT, histological analysis of tumors in the mice of four different groups were carried out after 14 days post‐treatment (Figure 6f). As demonstrated by the TUNEL imaging assay, mice treated with MFSPs@mSiO_2_‐ZnPC + 980 nm laser irradiation presented obvious apoptotic cells in the tissue. Besides, the H&E stained images of major organs (heart, liver, spleen, lung, and kidney) from mice intravenously injected with MFSPs showed the histological features were largely similar with no apparent organ damage or significant abnormalities observed compared to samples from healthy control (Figure 6g). Taken together, these results indicated that the MFSPs@mSiO_2_‐ZnPC had no long‐term toxicity, hopefully as a biocompatible PDT agent.

## 8 Conclusion

Hence, a very simple microemulsion based method was developed to synthesize UCNPs/Fe_3_O_4_ superparticles aimed for in vivo magnetically targeted NIR‐II imaging and PDT. The microemulsion based method could be used to produce UCNPs‐based sphere superparticles from any monodispersed nanoparticles, and the size of the superparticles is regulated by the organic solvent, water, and surfactant contents. Up to now, achieving precise size and size distribution control of the superparticles still meets some difficulties due to the inherent properties of microemulsion. In addition, the UCNPs’ fluorescence efficiency inside superparticles may not be as high as the UCNPs on the surface since the upconversion emission inside is hard to go outside. The photosensitizer (ZnPC)‐incorporated mesoporous silica shell was coated on superparticles to construct an imaging‐guided PDT multifunctional nanoplatform, in which 808 nm excitation was for real‐time NIR‐II imaging for diagnosis, and the upconversion luminescence upon 980 nm excitation was for on‐demand photosensitizer activation. Tumors in mice were markedly eliminated after MFSPs injection with the help of 980 nm laser irradiation and magnetic targeting. Our results suggested that UCNPs/Fe_3_O_4_ superparticles had a great potential to be valuable for diagnosis and treatment in the future.

## Conflict of Interest

The authors declare no conflict of interest.

## Supporting information

Supporting Information 1

Supporting Information 2

## Data Availability

The data that support the findings of this study are available in the supplementary material of this article.
