# Supplementary material for: Multifunctional Superparticles for Magnetically Targeted NIR‐II Imaging and Photodynamic Therapy
Source: Adv Sci (Weinh). 2022 Nov 22;10(2):2203669. doi: 10.1002/advs.202203669 (PMC9839852; doi:10.1002/advs.202203669)
Supplement: Supplementary file 1 — Supporting Information 1 [file ADVS-10-2203669-s001.pdf]

ADVANCED SCIENCE
Open Access

Supporting Information

for *Adv. Sci.*, DOI 10.1002/advs.202203669

Multifunctional Superparticles for Magnetically Targeted NIR-II Imaging and Photodynamic Therapy

Yilin Liu, Yuan Liang, Pengpeng Lei, Zhen Zhang* and Yongming Chen*

Supporting Information

Multifunctional Superparticles for Magnetically Targeted NIR-II Imaging and Photodynamic Therapy

Yilin Liu[#], Yuan Liang[#], Pengpeng Lei[*], Zhen Zhang[*], Yongming Chen

Miss Y. L. Liu, Dr. Z. Zhang, Prof. Y. M. Chen

School of Materials Science and Engineering, Sun Yat-sen University, Guangzhou 510275, People's Republic of China

Email: zhangzh379@mail.sysu.edu.cn

Mr. Y. Liang, Dr. P. P. Lei

State Key Laboratory of Rare Earth Resource Utilization, Changchun Institute of Applied Chemistry, Chinese Academy of Sciences, 5625 Renmin Street, Changchun 130022, China

Email: leipp@ciac.ac.cn

Mr. Y. Liang

University of Science and Technology of China, Hefei 230026, China

Ganjiang Innovation Academy, Chinese Academy of Sciences, Ganzhou, Jiangxi 341000, China

[#] These authors contributed equally.

Materials

Cyclohexane, 1-octadecene (ODE, 90%), Oleic acid (OA, 90 %), YCl_3•$6H_2O$ (99.9%), $ErCl_3$•$6H_2O$ (99.9%), $YbCl_3$•$6H_2O$ (99.9%), $LuCl_3$•$6H_2O$ (99.9%), NaOH (98+%), NH_4F (98+%), Sodium dodecyl sulfate (SDS), Cetyltrimethylammonium bromide (CTAB), Tetraethyl orthosilicate (TEOS, (98%), (3-aminopropyl) triethoxysilane (APTES, 99%), Zinc phthalocyanine (ZnPC), 4-(Phenylazo)benzoyl chloride (97%), Tetramethylammonium nitrate (98+%), Dimethyl sulfoxide (DMSO, 99%), Triethylamine (99.5%) and Paclitaxel (97%) were purchased from Sigma-Aldrich and used as received without further purification. 4′,6-Diamidino-2-phenylindole (DAPI) and the ROS assay kit, Cell Counting Kit-8, Cell culture medium, fetal bovine serum (FBS), Penicillin-streptomycin, Trypsin-EDTA, and the CellMask™ green plasma membrane stain were purchased from Thermo Fisher Scientific.

Physical characterization

Upconversion luminescence spectra were recorded by an FS-5 fluorescence spectrophotometer (Edinburgh Instruments) equipped with NIR continuous wave (CW) lasers with emitting wavelengths at 980 and 808 nm. The DLS size distribution was detected by a Malvern Zetasizer Nano Series (Malvern Instruments Ltd, Worcestershire, UK). Transmission electron microscopy (TEM) was recorded by a JEOL 2010F transmission electron microscope operating at an acceleration voltage of 200 kV and a JEM-1400Plus transmission electron microscope at 120 kV of accelerating voltage.

Methods

Synthesis of core UCNPs ($NaYF_4$: 20%Yb, 2%Er)

Core UCNPs were synthesized by a standard solvent thermal method. Typically, a total amount of 0.78 mmol YCl_3, 0.2 mmol $YbCl_3$, and 0.02 mmol $ErCl_3$ aqueous solution was added to a 100 mL flask. After evaporation of water at 120 ºC, a white dry

precipitate was formed. Subsequently, 6 mL OA and 15 mL ODE were added and the mixture was heated to 156 ºC to form rare-earth ions-oleate complexes until no more precipitate remained in the solution. The resulted yellow clear solution was then cooled to room temperature and a methanol solution (5 mL) containing NH_4F (4 mmol) and NaOH (2.5 mmol) was subsequently added. After that, the reaction temperature was raised to 120 ºC until the full removal of methanol. Next, the solution was degassed to completely remove residual methanol and oxygen. Subsequently, the resulted solution was raised to 304 ºC and lasted for 50 mins under an argon environment for the crystal growth. Then, the resulted solution was added with equivoluminal acetone and precipitated down under centrifugation at 8000 rpm for 10 mins, and further washed by acetone/cyclohexane mixed solution (volume ratio is 1:1) three times. The resulted products were finally dispersed in 20 mL cyclohexane for further usage.

Synthesis of core-shell structured UCNPs ($NaYF_4$: 20%Yb, 2%Er@$NaLuF_4$: 25%Y)

Core-shell structured UCNPs were prepared through epitaxial growth. The as-prepared core UCNPs in cyclohexane were employed as seeds for shell growth. In a typical process, an aqueous solution of 0.25 mmol YCl_3 and 0.75 mmol $LuCl_3$ were added into a 100 mL flask for every 1 mmol core UCNPs. Similarly, after fully evaporating the water, 6 mL OA and 15 mL ODE was added and the mixture was heated to 156 ºC to form lanthanide-oleate complexes. Upon the mixture became a clear solution, the as-prepared core UCNPs dispersed in 20 mL cyclohexane were added, and the resulted mixture was kept at 120 ºC for complete evaporation of cyclohexane. Subsequently, upon cooling to room temperature, 5 mL of methanol containing NH_4F (4 mmol) and NaOH (2.5 mmol) was added to the solution, followed by the removal of methanol at 120 ºC. Next, the reaction was degassed three times to remove the residual methanol

and oxygen, and the solution temperature was subsequently raised to 304 ºC under kept stirring in an argon atmosphere for 1.5 hours. The resulted nanoparticles were precipitated down after the addition of equivoluminal acetone under 8000 rpm centrifugation for 10 mins, followed by washing three times, and finally dispersed in 20 mL cyclohexane for further usage. The quantum yields of UCNPs tested is about 0.2% at a power density of 2 W cm^{-2}.

Synthesis of Fe_3O_4 nanoparticles

Briefly, iron-oleate complex (3.60 g), 1-octadecene (25.30 mL) and oleic acid (0.57 g) were added into a 100 mL flask. The temperature of the mixture was raised to 320 ºC and kept stirring under an argon atmosphere for 30 mins. The black-brown mixture was cooled to room temperature. The resultant nanoparticles were precipitated down after the addition of ethanol under 3000 rpm centrifugation for 5 mins, washed with acetone/cyclohexane mixed solution (volume ratio is 2:1), and finally dispersed in cyclohexane for further usage.

Synthesis of UCNPs/Fe_3O_4 MFSPs

A micro-emulsion based method was utilized to co-assemble UCNPs and Fe_3O_4 nanoparticles. Firstly, well-dispersed OA-capped UCNPs and Fe_3O_4 nanoparticles (total concentration is 5 mg/mL, and the weight ratio is 1:1) were mixed in the oil phase (cyclohexane, 1 mL), and the mixture was further added by 10 mL surfactant aqueous solution (SDS, 0.6 mg/mL). A stable oil/water emulsion mixture was obtained via vigorous stirring and sonication. Subsequently, the milk-like mixture was heated to 70 °C for at least 4 hours to fully evaporate the low-boiling cyclohexane, until the mixture turned clear, transparent aqueous solution. During the removal of cyclohexane, the droplets shrink and the two types of nanoparticles within the emulsion droplets (cyclohexane) get concentrated and pack closely with each other through hydrophobic

Van der Waals interactions of OA on the surface of UCNPs and Fe_3O_4 nanoparticles, thereby assembling to form UCNPs/Fe_3O_4 superparticles.

Synthesis of mesoporous silica-coated MFSPs

10 µL APTES was added to 10 mL 0.5 mg/mL MFSPs aqueous solution, which was stirred for 30 mins to ensure that the MFSPs could adsorb enough positively-charged APTES molecules for further effective TEOS absorption. Subsequently, 10 mL CTAB aqueous solution (3.5 mg/mL) and 2 mL ethanol were added to the above MFSPs solution under continuous stirring for 30 mins at 60 °C. Then, 100 µL 33% ammonium hydroxide solution was added into the mixture, followed by 50 µL TEOS added dropwise and the above solution was kept stirring for 2 hours. After washing twice with equivoluminal acetone and methanol mixture, the resulted MFSPs were dispersed in 10 mL ethanol. From TEM in Figure S3, the first layer of silica with about 3.5 nm thickness was successfully coated. Then, the same procedures were repeated to coat the second silica shell: MFSPs were treated with 10 mL water containing 35 mg CTAB and 2 mL ethanol under stirring for 30 mins at 60 °C, followed by 50 µL 33% ammonium hydroxide solution and 25 µL TEOS.

To make the silica with mesoporous pores, the two-layer silica-coated MFSPs dispersed in 10 mL ethanol was further added with 10 mL ethanol dissolved with 300 mg tetramethylammonium nitrate. Then, the mixture was refluxed at 60 °C for 2 hours. After that, the resulted nanoparticles were washed with equivoluminal acetone and ethanol twice and at last dispersed in 10 mL ethanol.

Loading photosensitizers into MFSPs@mSiO_2

To load the photosensitizer, zinc phthalocyanine (ZnPc), into the mesoporous pores, 5 mg MFSPs@mSiO_2 were soaked with 0.1 mg ZnPC in 2 mL DMSO with continuous stirring for 24 hours at room temperature. The resulted MFSPs@mSiO_2-ZnPc were

then precipitated and washed by the deionized water to remove the nonabsorbed ZnPc molecules, and finally redispersed in water. To evaluate the loading capacity, the UV-vis absorption spectra of the original ZnPC solution and supernatant were measured.

Release measurement of ZnPc from MFSPs@mSiO_2

1 mg MFSPs@mSiO_2-ZnPc was dispersed in either 1 mL deionized water, 1× PBS, and DMSO for 24 hours. Subsequently, the MFSPs@mSiO_2-ZnPc were precipitated by 10000 rpm centrifugation for 10 mins, and their supernatants were respectively collected. To determine the quantity of released photosensitizers in the solution, the UV-vis absorbance of each supernatant solution was measured.

Detection of ROS

A DPBF probe was employed for the detection of ROS. Typically, 2 mL MFSPs@mSiO_2-ZnPc aqueous solution was added by 10 mmol/L DPBF dissolved in 10 μL ethanol, which was then transferred to a cuvette for UV-vis absorption test, which was protected from room light during the test. Then, the solution was irradiated by lasers, either 980 nm (0.8 W/cm^2) or 808 nm (0.2 W/cm^2) for 30 mins. The UV-vis absorption intensity at 417 nm was recorded every 5 mins. For the control groups in the absence of MFSPs@mSiO_2-ZnPc or 980 nm laser irradiation, the absorption intensity at 417 nm was also recorded using the same setup for a fair comparison.

Loading FITC into MFSPs@mSiO_2-ZnPC

To load the FITC into the mesoporous pores, 10 mg MFSPs@mSiO_2-ZnPC were soaked with 1.0 mg FITC in 2 mL aqueous solution with continuous stirring for 4 hours at room temperature. The resulted FITC-labelled MFSPs@mSiO_2-ZnPc were then precipitated and washed by the deionized water to remove the nonabsorbed FITC molecules, and finally redispersed in water. To evaluate the loading capacity, the

UV-vis absorption spectra of the original FITC solution and supernatant were measured (about 4.67 wt%).

Cell culture

4T1 cells were grown in the DMEM culture medium, supplemented with 10% FBS (Life Technologies), 100 μg/mL of streptomycin, and 100 units/mL of penicillin. The cells were incubated at 37 °C in a humidified, 5% (v/v) CO_2 atmosphere.

Cellular uptake of MFSPs@mSiO$_2$-ZnPC

4T1 cells were seeded at a concentration of 1.5×10^5 cells per dish for 24 hours to allow effective cell attachment. Subsequently, cells were incubated with 100 μg/mL MFSPs@mSiO$_2$-ZnPc for 24 hours at 37 °C. Then, cells were fixed in 4% paraformaldehyde for 15 mins at room temperature, followed by washing with PBS for three times. MFSPs@mSiO$_2$-ZnPC stained with fluorescein isothiocyanate (FITC) were cultured with 4T1 cells, and cells were stained using 4,6-diamino-2-phenyl indole (DAPI) in different time periods of culture for 10 mins, followed by fixation using tissue fixative, and finally washed and placed under a fluorescent inverted microscope for photographs.

Cytotoxicity and PDT *in vitro*

The CCK-8 assay was carried out to determine the cytotoxicity and PDT effects. The 4T1 cells were seeded in 96-well microplates at a concentration of 8000 cells per well for 24 hours. Subsequently, 100 μL MFSPs@mSiO$_2$-ZnPc with different concentrations (0, 15.625, 31.25, 62.5, 125, 250, 500, and 1000 μg/mL) were co-cultured with cells for 24 hours. After washing twice with PBS, 4T1 cells in each well were incubated with 10 μL CCK-8 assay for 1 hour and the microplate reader was employed to record the absorbance at 450 nm for the cell viability determination.

For the *in vitro* PDT experiment, similarly, the cells were incubated with different concentrations of MFSPs@mSiO_2-ZnPc (0, 15.625, 31.25, 62.5, 125, 250, 500, and 1000 μg/mL) for 24 hours and then washed twice with PBS before being exposed to 980 nm laser irradiation (0.8 W/cm^2) for 10 mins. Subsequently, the cells after irradiation were incubated for another 24 hours. Similarly, 10 μL CCK-8 kit was added to each well and incubated for 1 hour at 37 °C, followed by the absorbance measurement at 450 nm for each well. Cell viability was then calculated as follows:

$$A_{laser}/A_{no-laser} \times 100$$

where $A_{laser}$is the absorbance of the laser-irradiated sample and $A_{no-laser}$ is the absorbance of the sample without laser irradiation.

Intracellular ROS detection

DCFH-DA was employed to determine the ROS generation of MFSPs@mSiO_2-ZnPC *in vitro*, which could be oxidized by ROS and emit green fluorescence. DCFH-DA solution (50 μM, 1 mL) was added to MFSPs@mSiO_2-ZnPC-treated 4T1 cells and the cells were further treated by 980 nm laser irradiation (0.8 W cm^{-2}) for different irradiation timings (0, 5, and 10 mins). Finally, the cells were washed with PBS three times, and the fluorescence from DCFH-DA inside cells was observed from an inverted fluorescence microscope.

Hemolysis assay

Blood samples obtained from mice were diluted with 10 mL PBS, from which red blood cells were precipitated by centrifugation at 1200 rpm for 10 mins. The red blood cells were washed completely and finally diluted with 10 mL PBS. Then, 200 μL diluted red blood cell suspension was mixed with 1 mL PBS (as a negative control), deionized water (as a positive control), and MFSPs@mSiO_2-ZnPC dissolved in PBS. After incubation for 4 hours at 37 °C, the suspensions were centrifuged at

12000 rpm for 10 mins, whose supernatants were subsequently added to a 96-well microplate and measured by a microplate reader for the absorbance intensity. The hemolysis percentage could be calculated by the following equation:

$$Hemolysis\ ratio\ (\%) = \frac{A_{sample} - A_{control(-)}}{A_{control(+)} - A_{control(-)}} \times 100\%$$

The live/dead staining *in vitro*

4T1 cells were seeded into 96-well microplates for 24 hours and followed by co-culture with different concentrations (0, 15.625, 31.25, 62.5, 125, 250, 500, and 1000 μg/mL) of MFSPs@mSiO$_2$-ZnPC for 2 hours. Subsequently, 4T1 cells treated by 980 nm laser irradiation (0.8 W cm^{-2}, 10 mins) were incubated for another 24 hours. The calcein AM/PI staining was used to depict the cell viability via the inverted fluorescence microscope.

***In vivo* NIR-II bioimaging**

Balb/c female mice (≈ 17 g) were purchased from Changsheng Biotechnology Co. Ltd. (320700000055905). All animal experiments have been approved by the Institutional Animal Care and Use Committee of Changchun Institute of Applied Chemistry (IACUC) (Grant No. 20220001).

For *in vivo* NIR-II bioimaging, 100 μL PBS solution containing 600 μg mL^{-1} MFSPs@mSiO$_2$-ZnPC was injected into each tumor-bearing mouse, whose NIR-II signals at 1000-1600 nm were chosen for the magnetic targeting and real-time NIR-II bioimaging *in vivo* and collected from anesthetized mice at different time intervals under 808 nm laser irradiation (0.2 W cm^{-2}).

PDT efficacy *in vivo*

Tumor-bearing mice were randomly divided into 6 groups, at least 5 mice in each group, as follows: (1) PBS, (2) 980 nm laser only, (3) MFSPs@mSiO$_2$-ZnPC (1000

μg mL^{-1}, 100 μL), (4) MFSPs@$mSiO_2$-ZnPC (1000 μg mL^{-1}, 100 μL) + magnetic field, (5) MFSPs@$mSiO_2$-ZnPC (1000 μg mL^{-1}, 100 μL) + 980 nm laser irradiation, and (6) MFSPs@$mSiO_2$-ZnPC (1000 μg mL^{-1}, 100 μL) + magnetic field + 980 nm laser irradiation. 8 hours after intravenous injection, the mice were anesthetized and the tumor sites were treated by 980 nm laser irradiation (0.8 W cm^{-2}, 10 mins), accordingly. During the experiments, the tumor size and body weight of each mouse were monitored every 2 days. Tumor volume V (mm^3) was calculated by the formula:

$$V = (length \times width^2)/2$$

Relative tumor volume was calculated as V/V_0, where V_0 is the corresponding tumor volume before treatment.

Subsequently, tumors were dissected and weighed to assess the PDT efficacy after 14 days. The tumor tissues were sliced to 4 μm thickness for hematoxylin and eosin (H&E) staining and TdT-mediated dUTP-biotin nick and labeling staining (TUNEL). The semi-quantification for the TUNEL assay was analyzed by Image-pro plus 6.0 (Media Cybernetics, Inc., Rockville, MD, USA).

Assessment of long-term toxicity

For the *in vivo* toxicity studies, healthy Balb/c mice were injected with 100 μL MFSPs@$mSiO_2$-ZnPC (1000 μg mL^{-1}). The mice without any treatment were used as the blank control. H&E staining of major organs (heart, liver, spleen, lung, and kidney) was performed after 28 days to observe histomorphology changes.

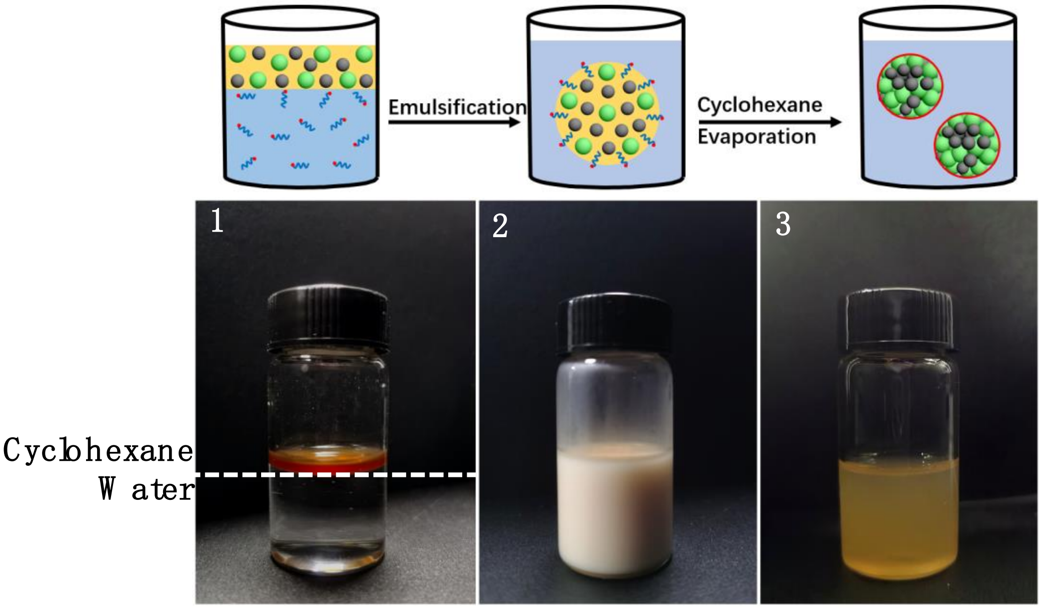

Figure S1. Schematic and photos of micro-emulsion based fabrication process of MFSPs: 1) 1 mL cyclohexane suspension of UCNPs and Fe_3O_4 (5 mg/mL, the weight ratio is 1:1) mixed with 10 mL water containing 6 mg SDS, forming immiscible two layers when mixed; 2) After vortex and sonication, the stable turbid micro-emulsion solution was obtained with the help of SDS as surfactants in aqueous solution; 3) UCNPs/F_3O_4 MFSPs solution was obtained after the evaporation of cyclohexane.

Table S1 Variations in zeta potentials after different surface modifications.

Sample	Averaged Zeta Potential (mV)
MFSPs	-24.6
MFSPs@mSiO$_2$	-17.4
MFSPs@mSiO$_2$-ZnPc	18.8
FITC	-16.6
FITC-labeled MFSPs@mSiO$_2$-ZnPC	-12.0

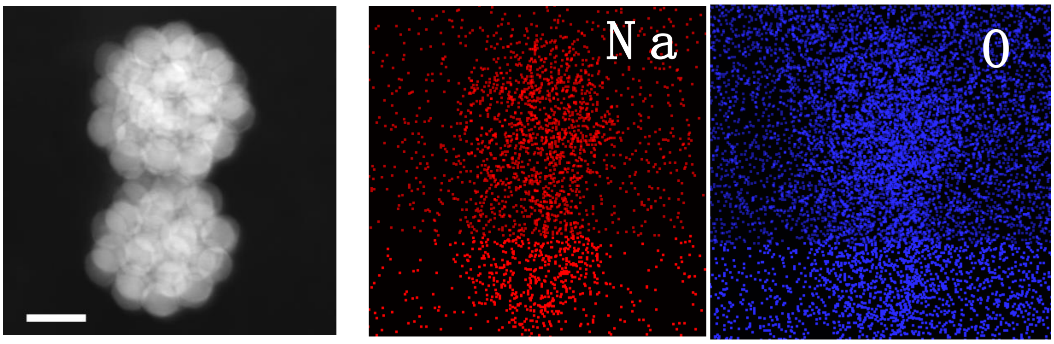

Figure S2. High-angle annular dark-field scanning transmission electron microscopy (HADDF-STEM) figures, and STEM elemental mapping of Na and O.

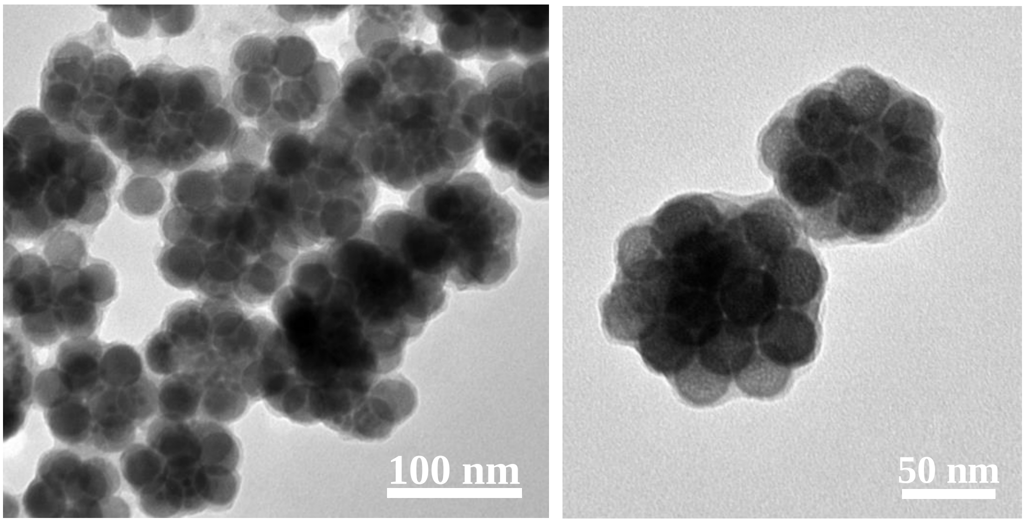

Figure S3. TEM images of MFSPs with a thin silica coating shell.

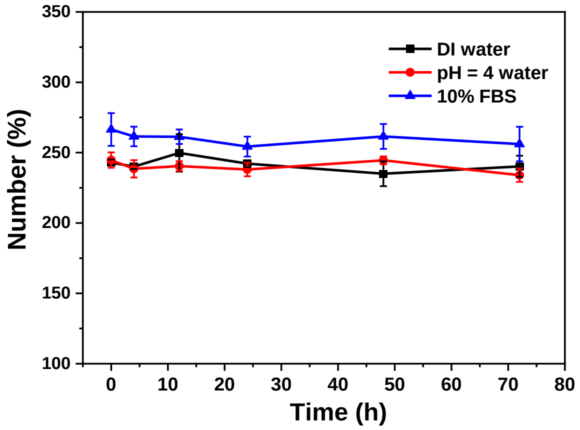

Figure S4. DLS data showing stability of MFSPs@mSiO_2 in DI water, pH = 4 water solution and 10% FBS conducted over 72 hours.

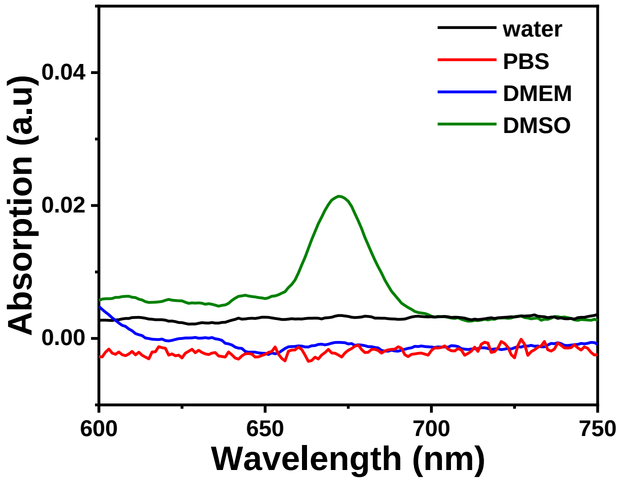

Figure S5. The UV-vis absorption of MFSPs@mSiO$_2$-ZnPC supernatants were collected by centrifugation after they were soaked in deionized water, PBS, cell culture (DMEM), and DMSO for 24 hours.

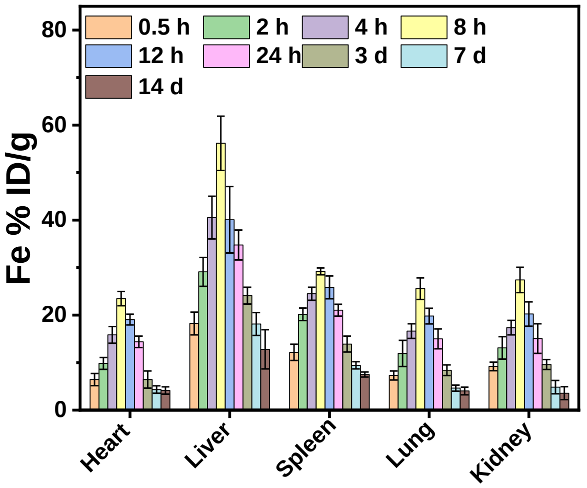

Figure S6. Percentage of injected dose of Fe in mice treated with MFSPs@mSiO$_2$-ZnPC at different time points from 0.5 hour to 14 days.

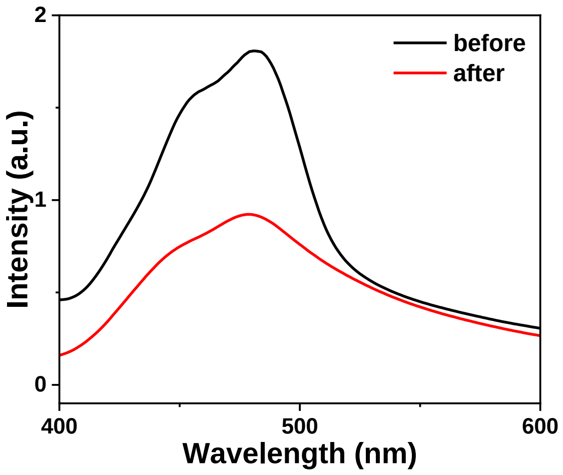

Figure S7. The UV-vis absorption of FITC aqueous solution (0.5 mg/mL, black line) and its supernatant after loading FITC into MFSPs@mSiO_2-ZnPc (red line).

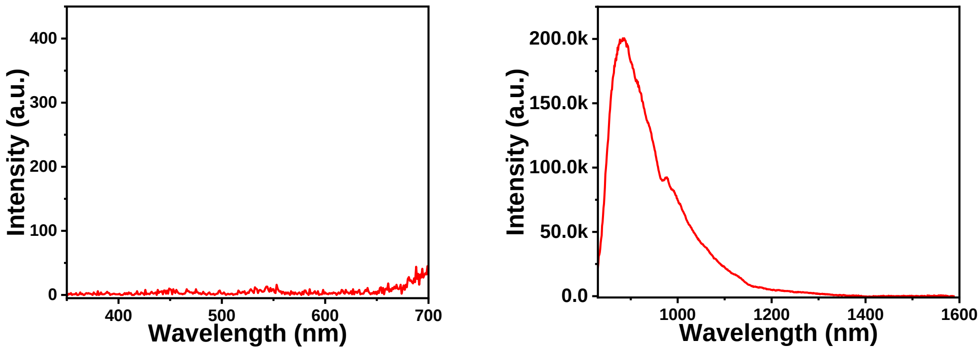

Figure S8. Emission spectrum of MFSPs@mSiO_2-ZnPC at 808 nm excitation.

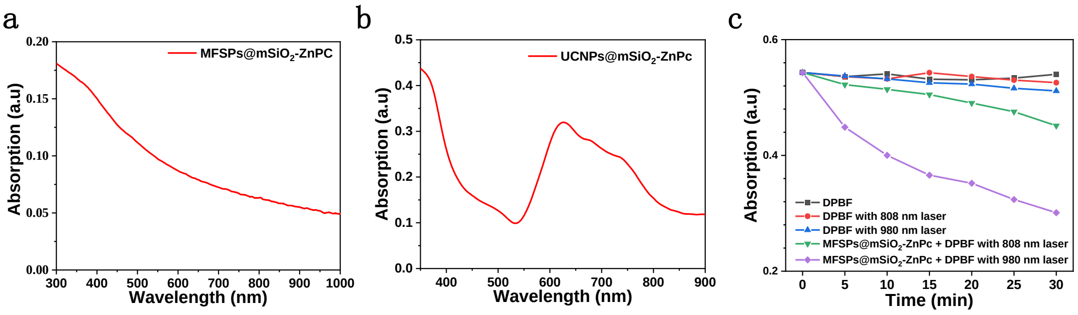

Figure S9. The UV-vis absorption spectra of MFSPs@mSiO_2-ZnPC (a) and UCNPs@mSiO_2-ZnPc (b) aqueous solution. c, Comparison of DPBF consumption for five groups (1) DPBF, (2) DPBF + 808 nm laser irradiation (0.2 W cm^{-2}), (3) DPBF + 980 nm laser irradiation (0.8 W cm^{-2}), (4) MFSPs@mSiO_2-ZnPC + DPBF with 808 nm

laser irradiation (0.2 W cm^{-2}), and (5) MFSPs@mSiO_2-ZnPC + DPBF with 980 nm laser irradiation (0.8 W cm^{-2}).

MFSPs@mSiO_2-ZnPC contains UCNPs, Fe_3O_4 nanoparticles, and ZnPC molecules within one superparticle, which has a broad UV-vis absorption spectrum from 300 to 1000 nm (Figure S9a). To eliminate the influence of Fe_3O_4 nanoparticles and identify the UV-vis absorption of ZnPC inside mesoporous silica directly, ZnPC-encapsulated mesoporous silica-coated UCNPs (UCNPs@mSiO_2-ZnPC) were synthesized, whose UV-vis absorption spectrum was shown in Figure S9b, with a broad wavelength ranging from 500 to 900 nm, different from that of ZnPC molecules in DMSO solution shown in Figure 2c. This indicates that the ZnPC absorption spectrum in mesoporous silica coating shell undergoes an obvious redshift, consistent with previous reports indicating that the absorption spectrum of ZnPC aggregates undergoes a redshift.[1-3] In our system, well-dispersed ZnPC molecules in DMSO were encapsulated into mesoporous silica pores and then the ZnPC-loaded superparticles were transferred to aqueous solutions. However, ZnPC is hydrophobic, so they tend to aggregate and confine inside the mesoporous silica pores. In addition, the UV-vis absorption peak at 600-700 nm of individual ZnPC molecules also remains in the absorption spectra in Figure S9b, indicating that individual ZnPC molecules and ZnPC aggregates co-exist inside mesoporous silica.

References:

1. Gao, D.; Wong, R. C. H.; Wang, Y.; Guo, X.; Yang, Z.; Lo, P. C., *Acta Biomater.* **2020,** *116*, 329-343.
2. Jin, H. G.; Zhong, W.; Yin, S.; Zhang, X.; Zhao, Y. H.; Wang, Y.; Yuan, L.; Zhang, X. B., *ACS Appl. Mater. Inter.* **2019,** *11*, 3800-3808.
3. Moon, H. K.; Son, M.; Park, J. E.; Yoon, S. M.; Lee, S. H.; Choi, H. C., *NPG Asia Mater.* **2012,** *4*, e12-e12.
