# Supplementary material for: Multifunctional Superparticles for Magnetically Targeted NIR‐II Imaging and Photodynamic Therapy
Source: Adv Sci (Weinh). 2022 Nov 22;10(2):2203669. doi: 10.1002/advs.202203669 (PMC9839852; doi:10.1002/advs.202203669)
Supplement: Supplementary file 2 — Supporting Information 2 [file ADVS-10-2203669-s002.ppt]

## Slide 1
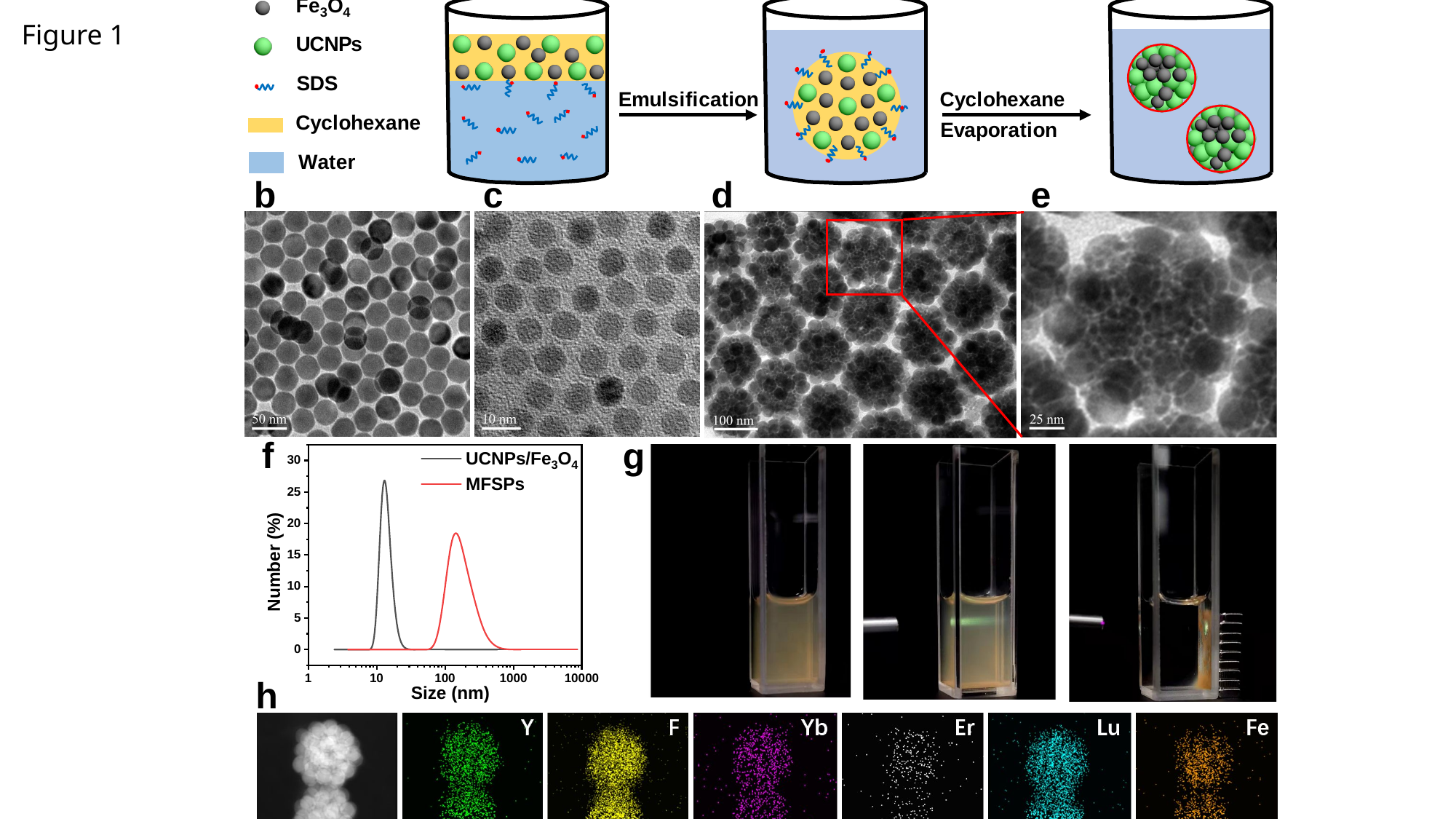

a
Figure 1
b
c
d
e
100 nm
f
g
h

## Slide 2
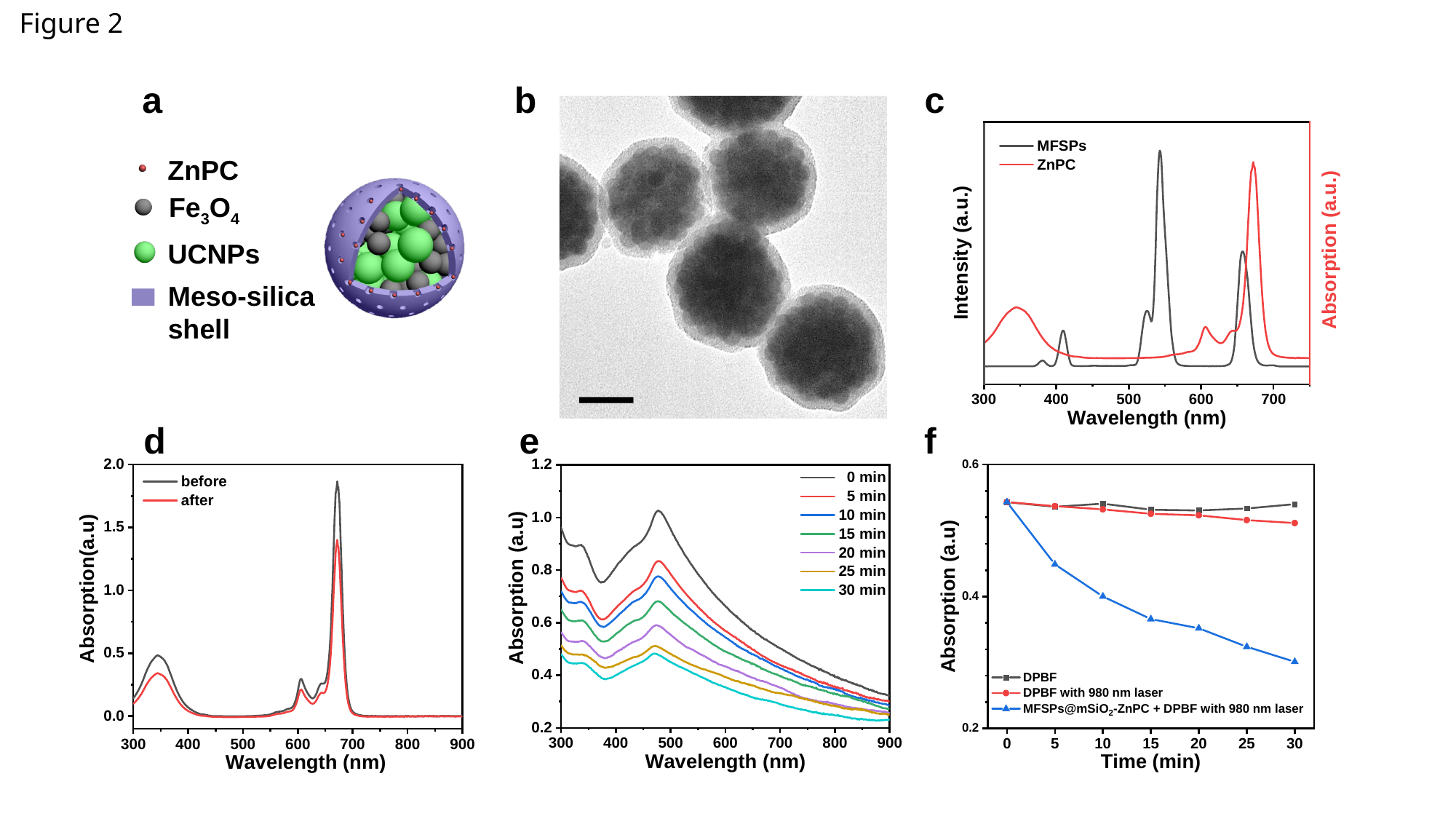

Figure 2
a
b
c
ZnPC
Fe3O4
UCNPs
Meso-silica
shell
d
e
f

## Slide 3
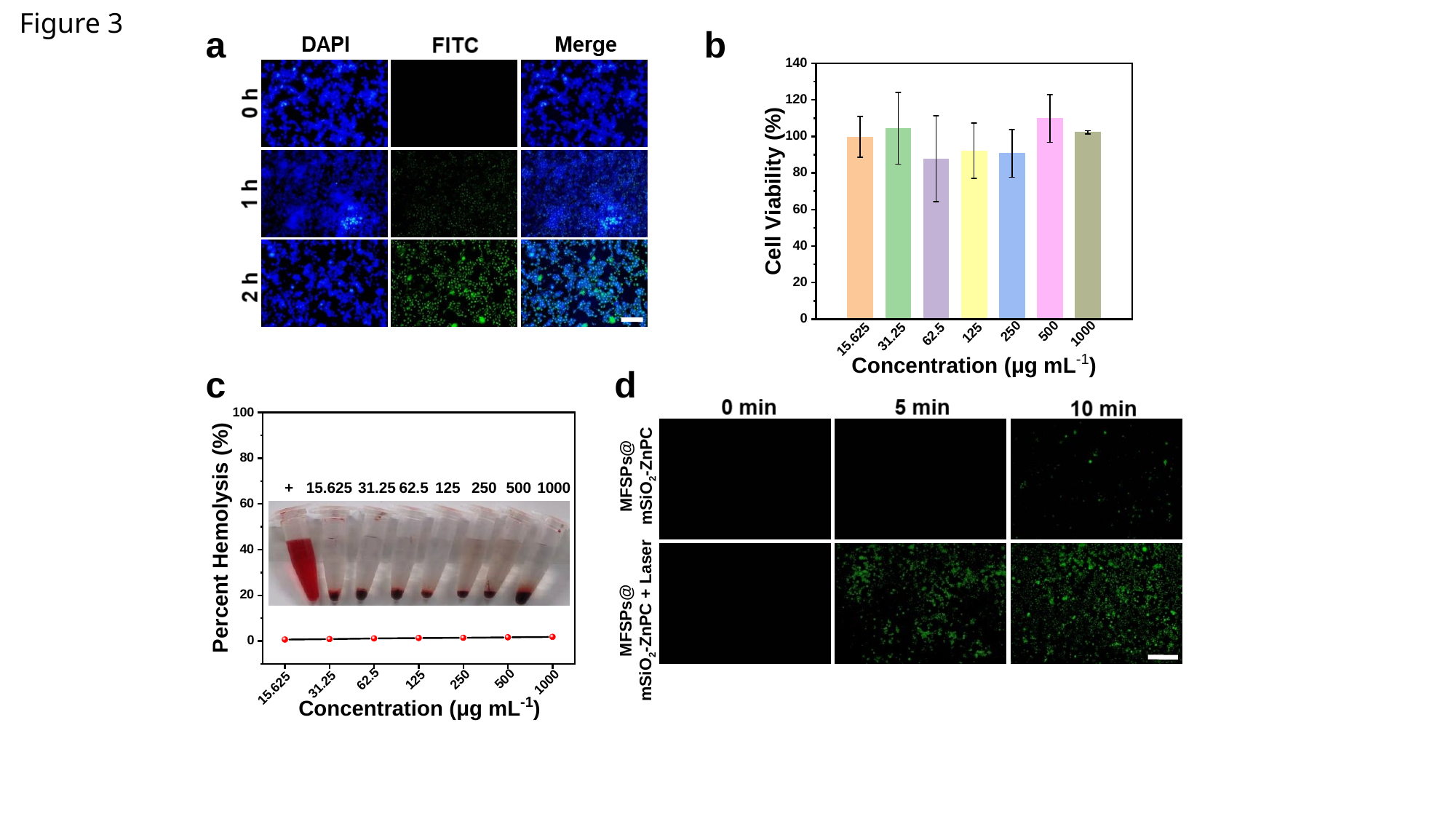

Figure 3
a
b
c
d
MFSPs@
mSiO2-ZnPC
MFSPs@
mSiO2-ZnPC + Laser
+
15.625
31.25
62.5
125
250
500
1000

## Slide 4
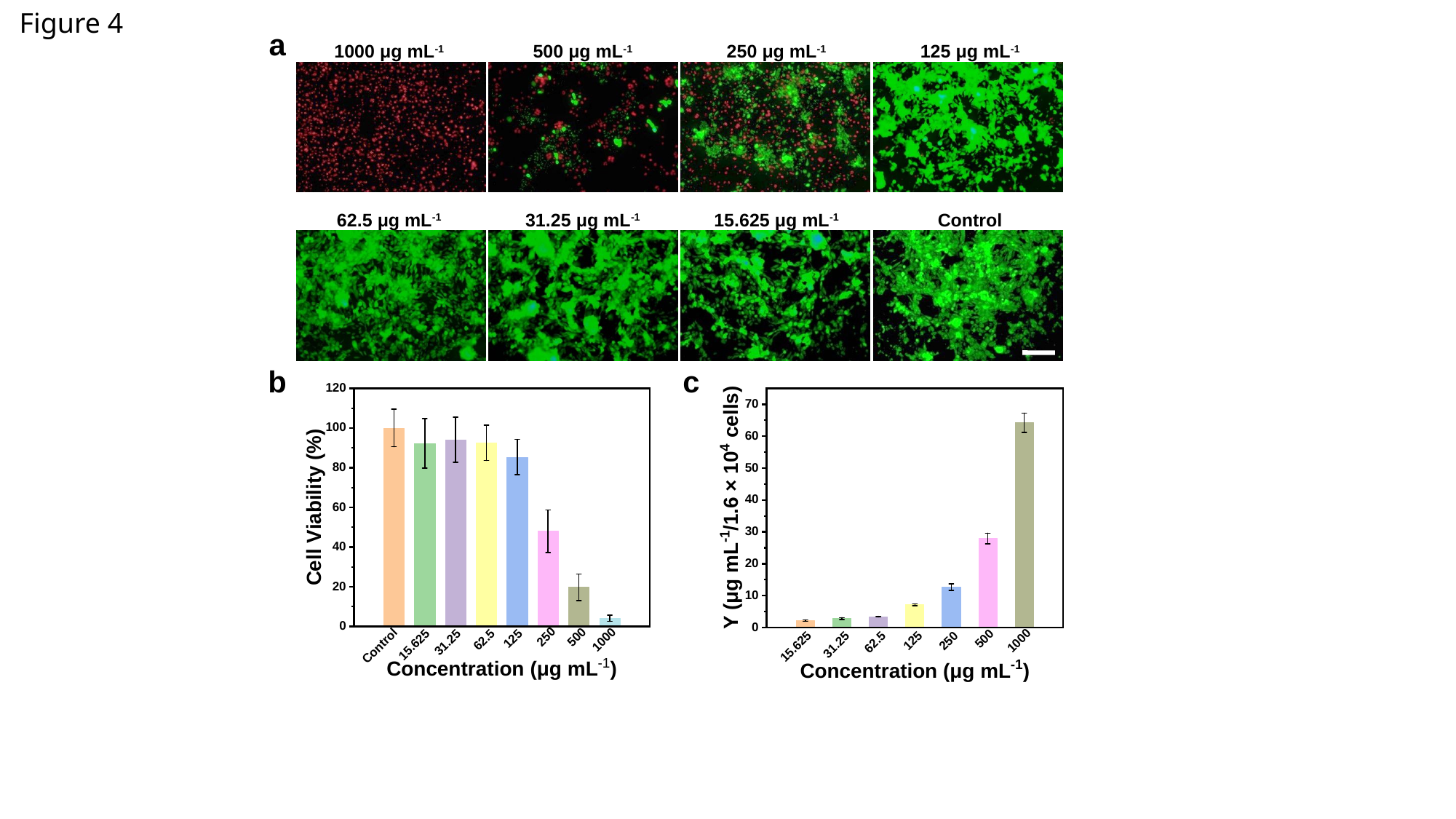

Figure 4
a
1000 μg mL-1
500 μg mL-1
250 μg mL-1
125 μg mL-1
62.5 μg mL-1
31.25 μg mL-1
15.625 μg mL-1
Control
b
c

## Slide 5
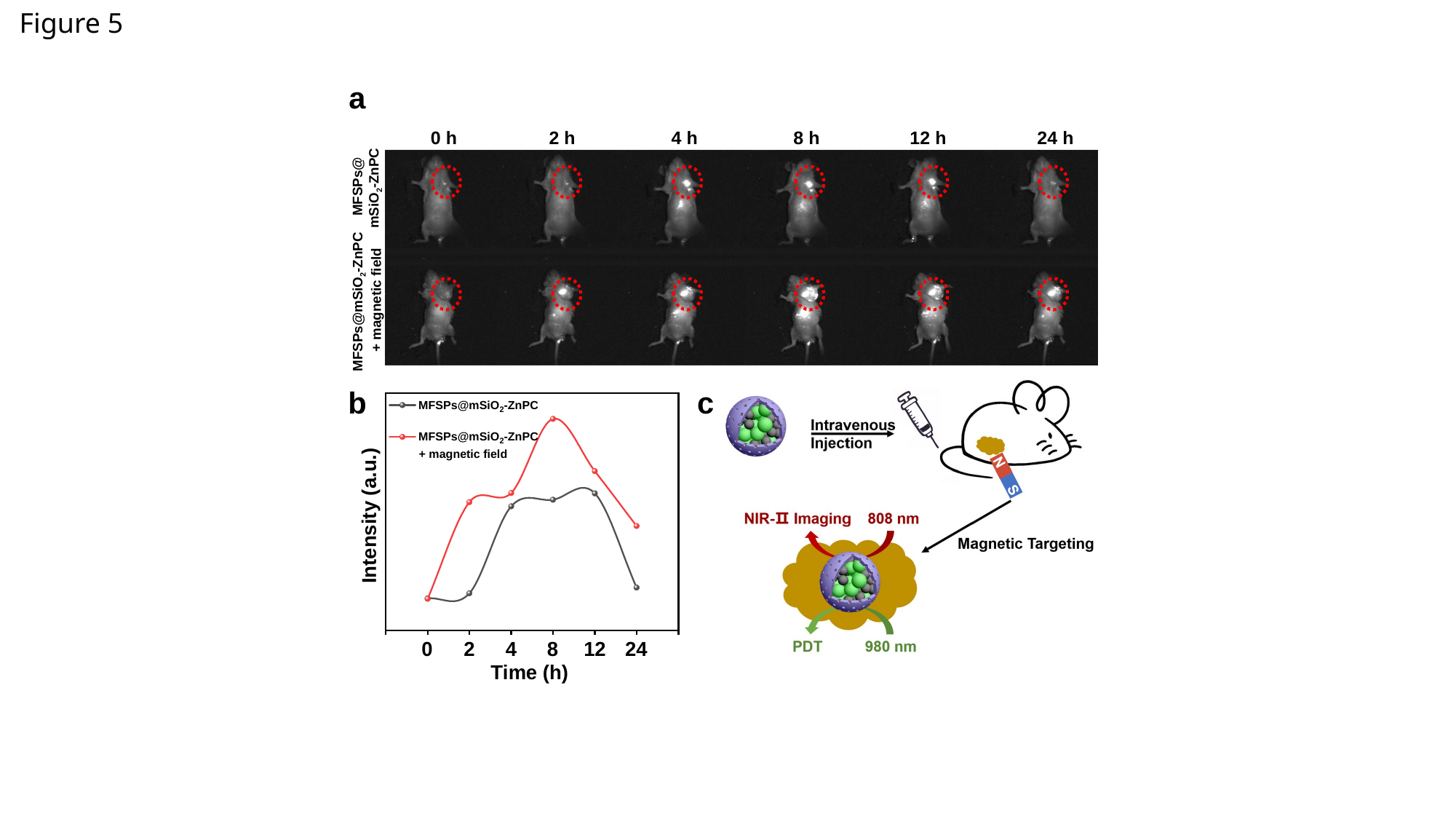

Figure 5
a
0 h
2 h
4 h
8 h
12 h
24 h
MFSPs@
mSiO2-ZnPC
MFSPs@mSiO2-ZnPC
+ magnetic field
b
c

## Slide 6
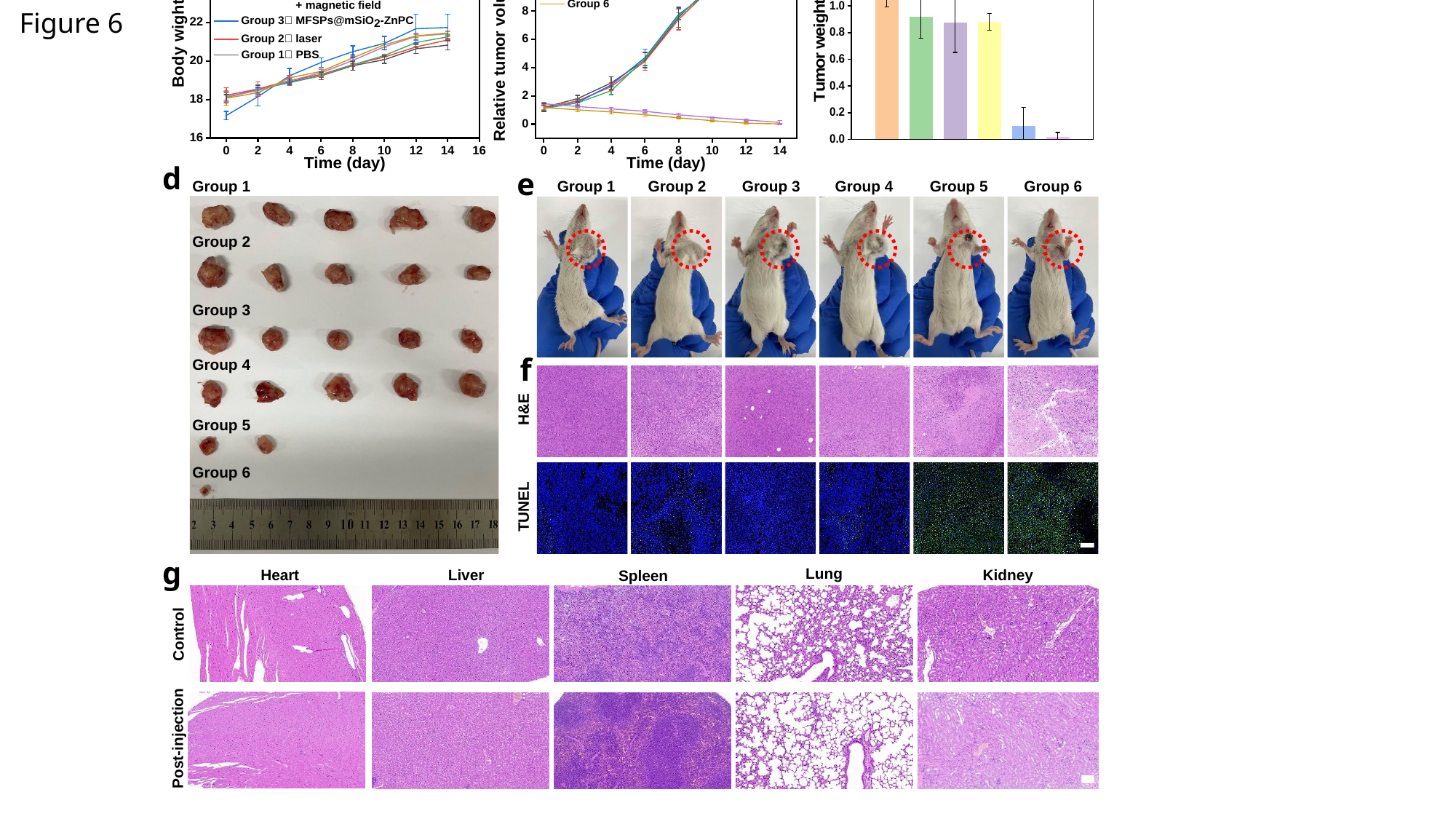

Figure 6
a
b
c
d
e
Group 1
Group 1
Group 2
Group 3
Group 4
Group 5
Group 6
Group 2
Group 3
f
Group 4
H&E
Group 5
Group 6
TUNEL
g
Lung
Heart
Liver
Kidney
Spleen
Control
Post-injection

## Slide 7
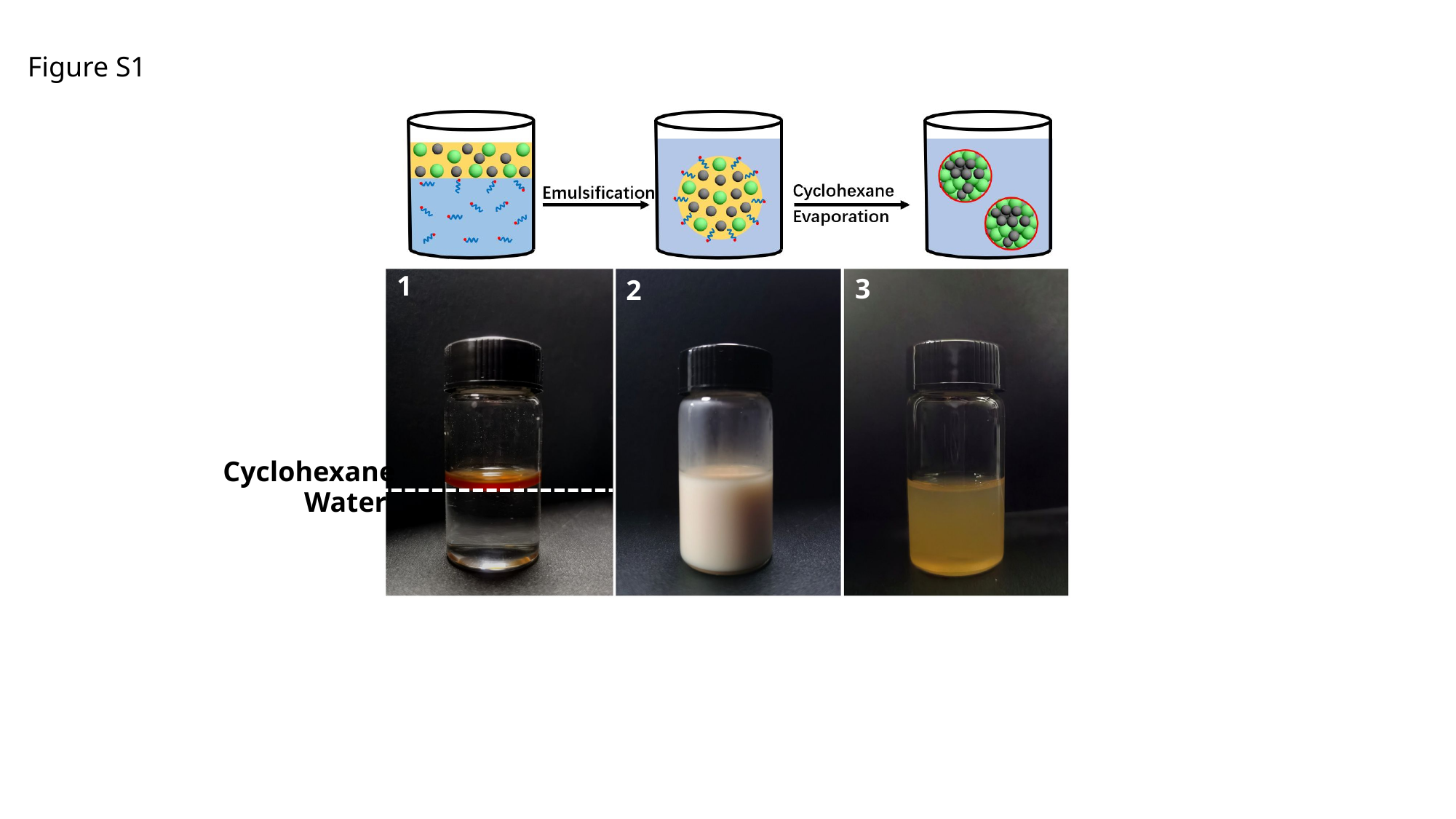

Figure S1
1
3
2
Cyclohexane
Water

## Slide 8
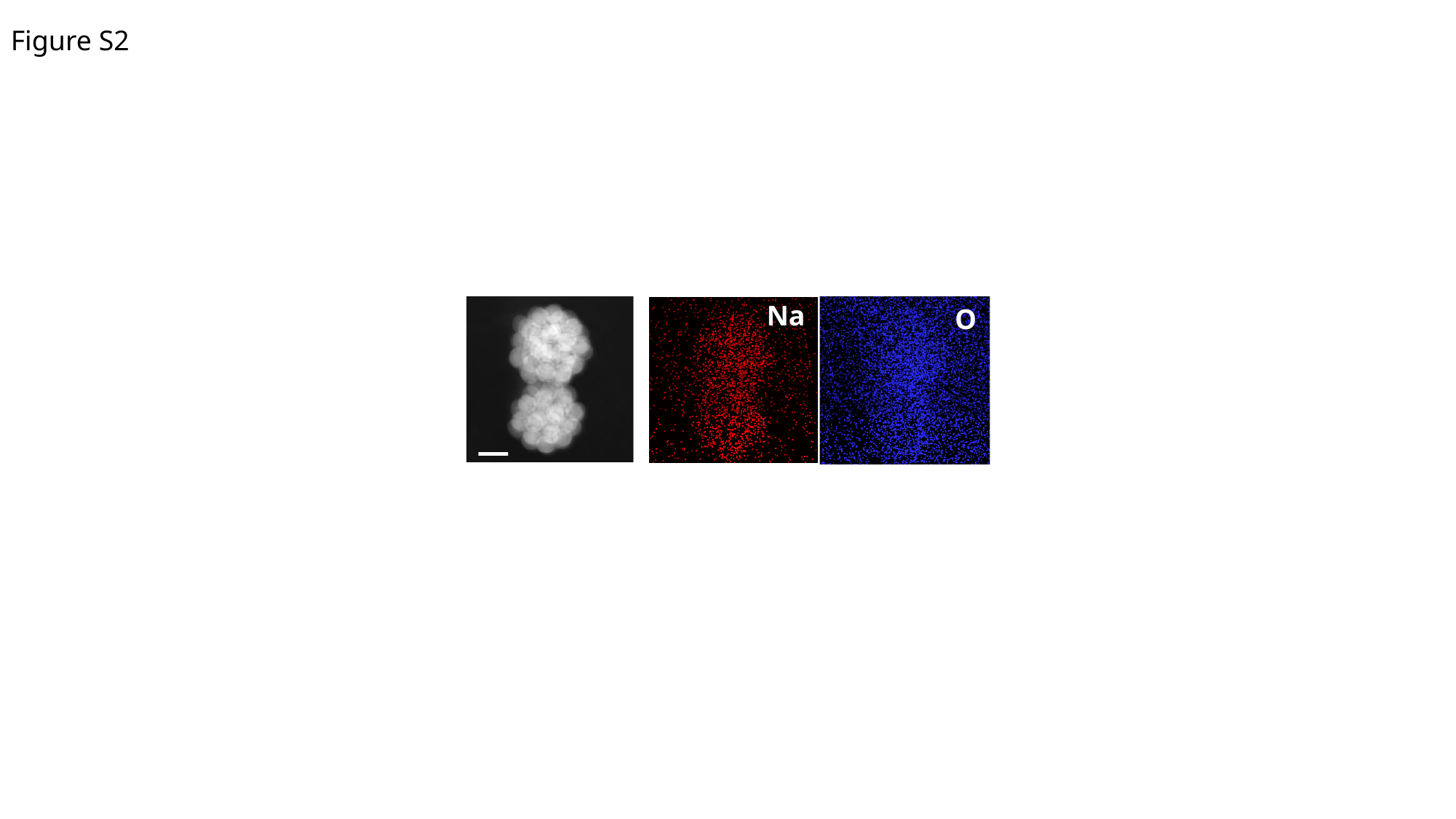

Figure S2
Na
O

## Slide 9
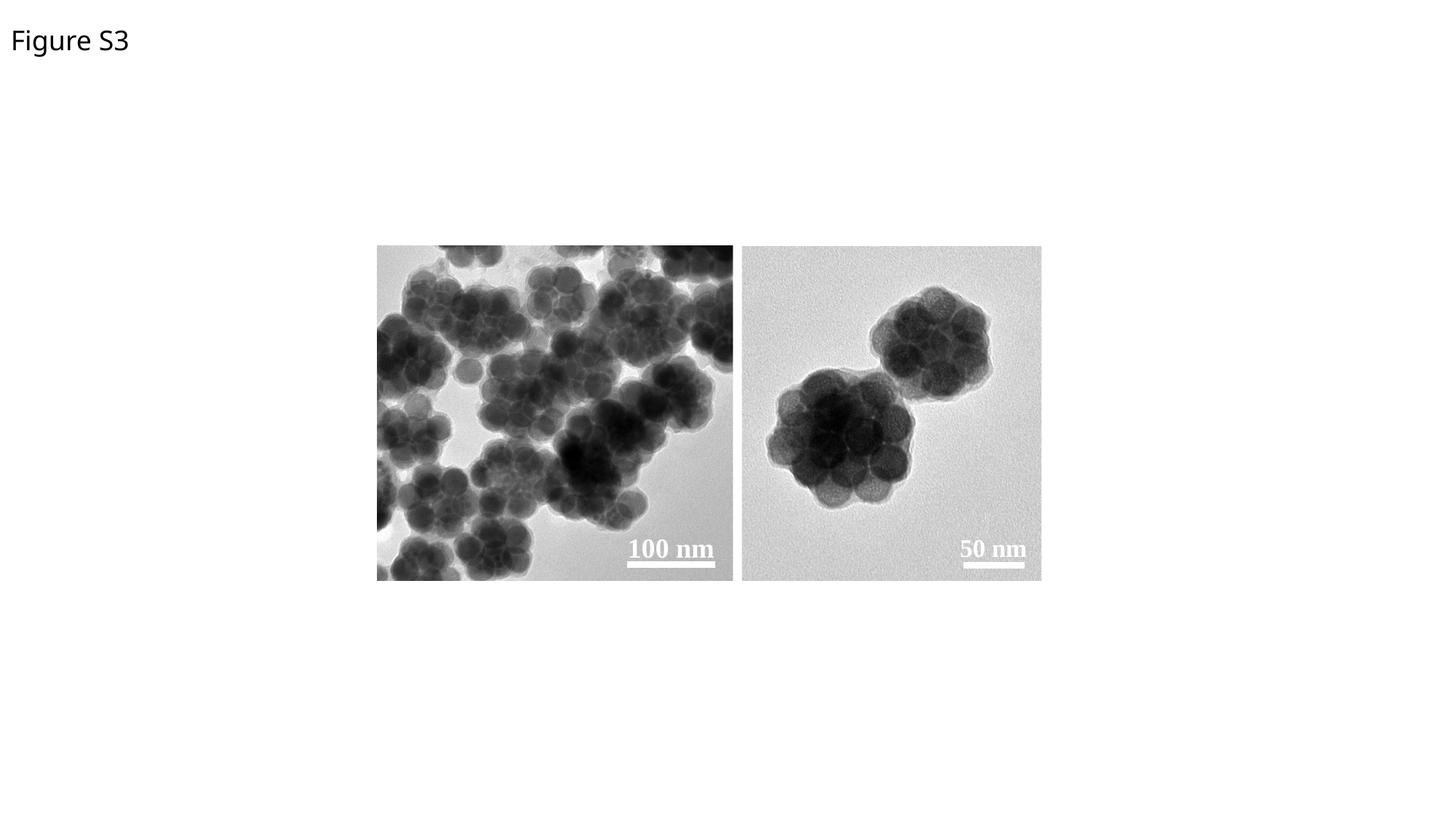

Figure S3

## Slide 10
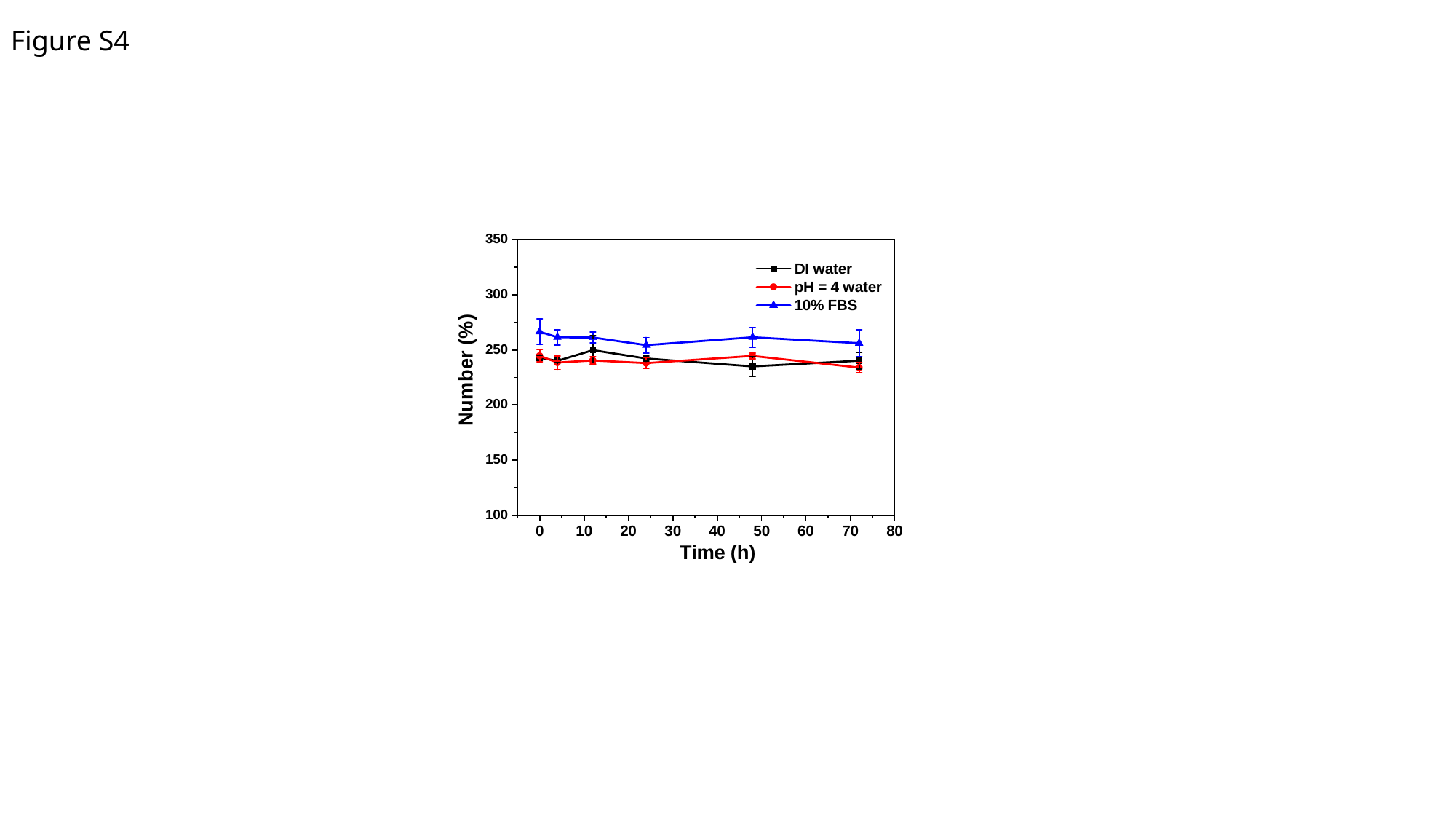

Figure S4

## Slide 11
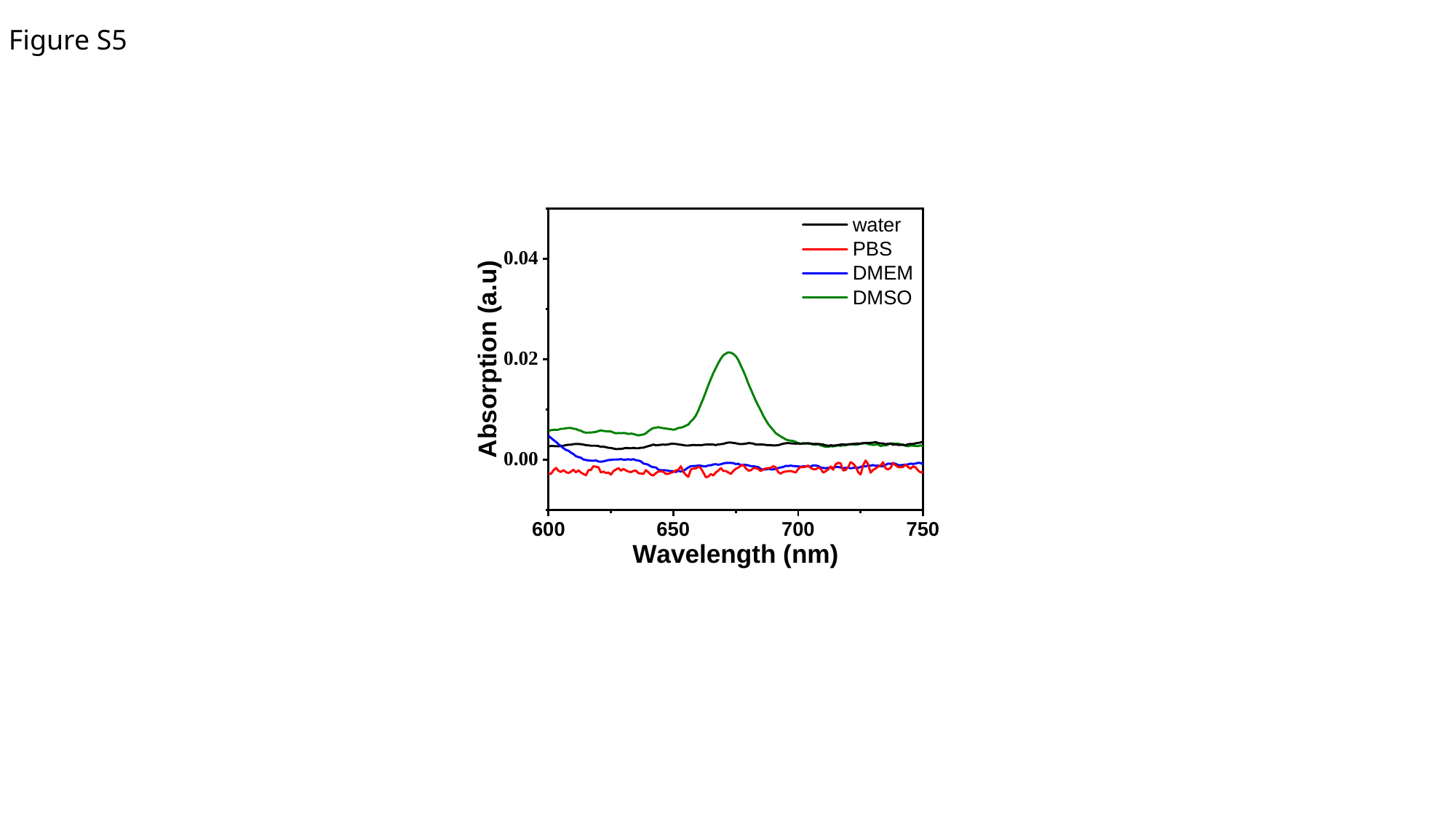

Figure S5

## Slide 12
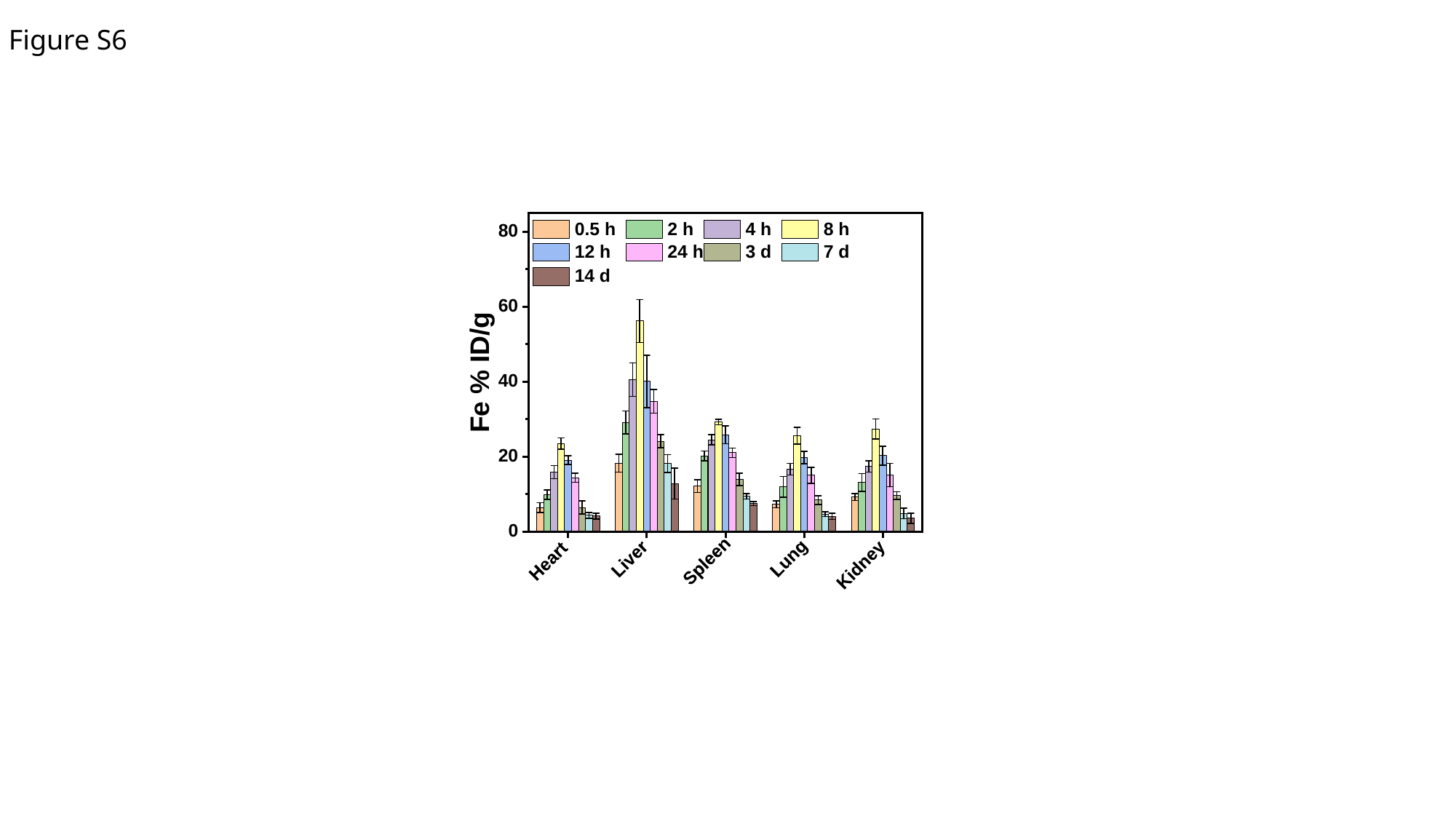

Figure S6

## Slide 13
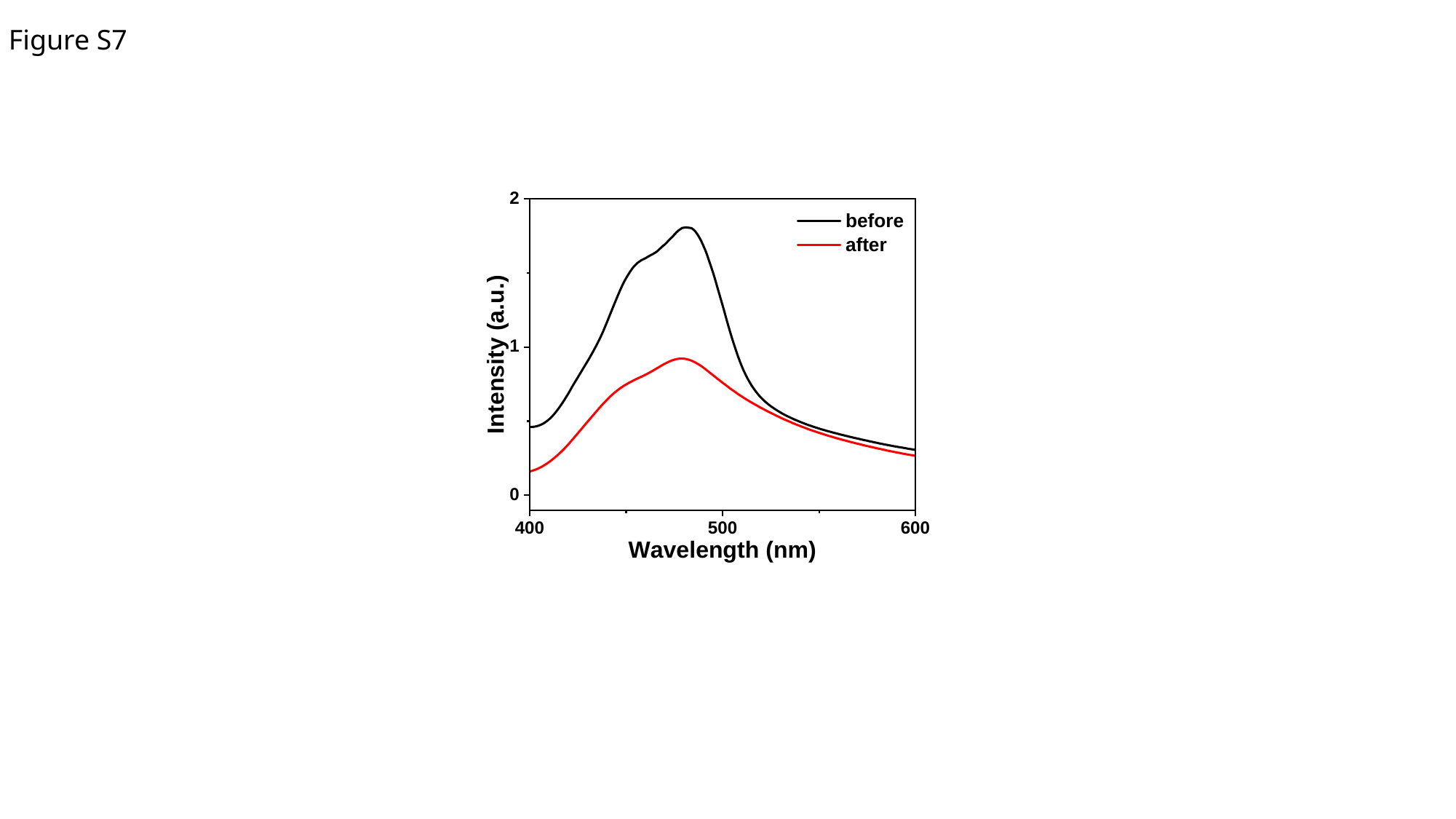

Figure S7

## Slide 14
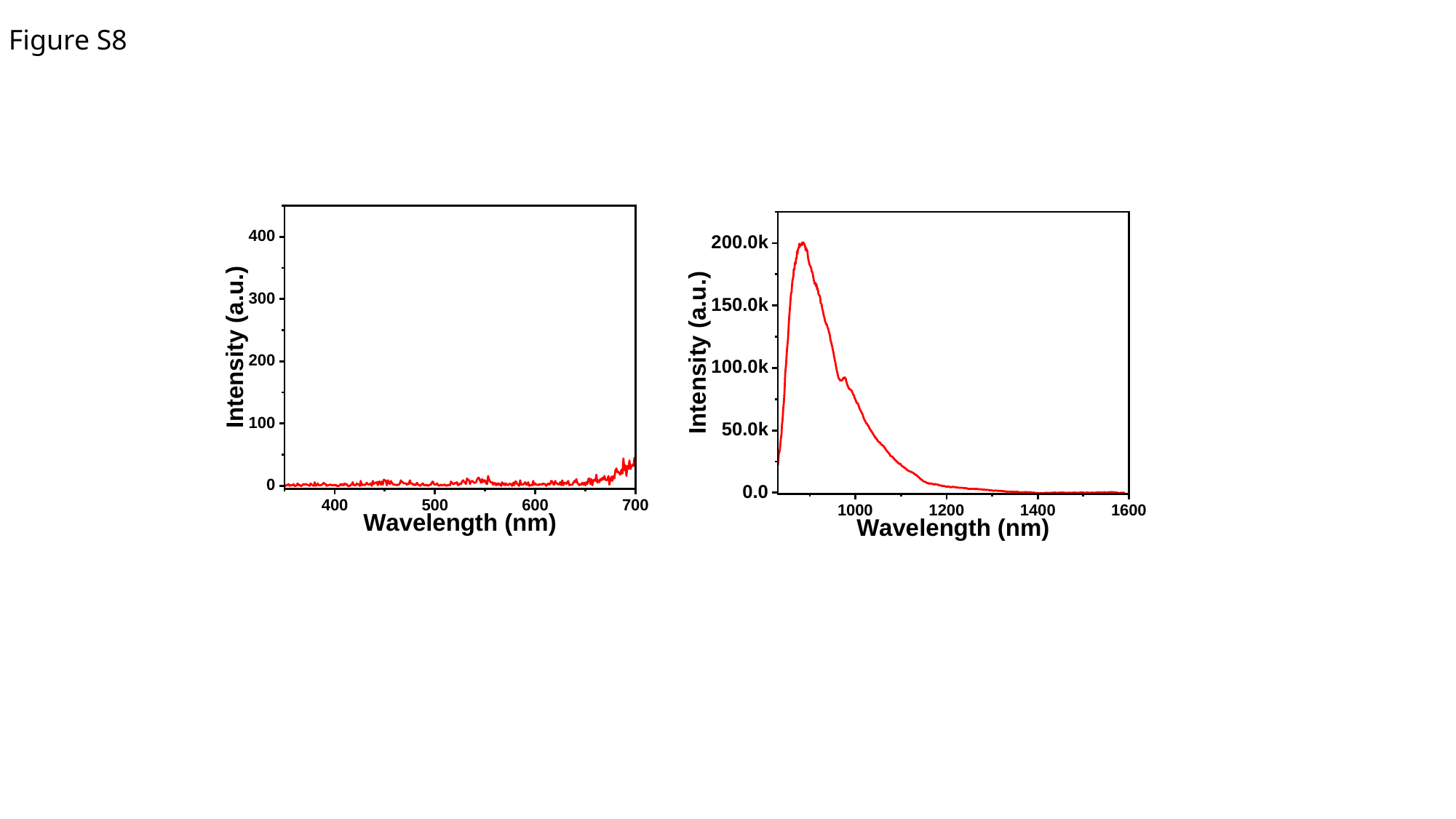

Figure S8

## Slide 15
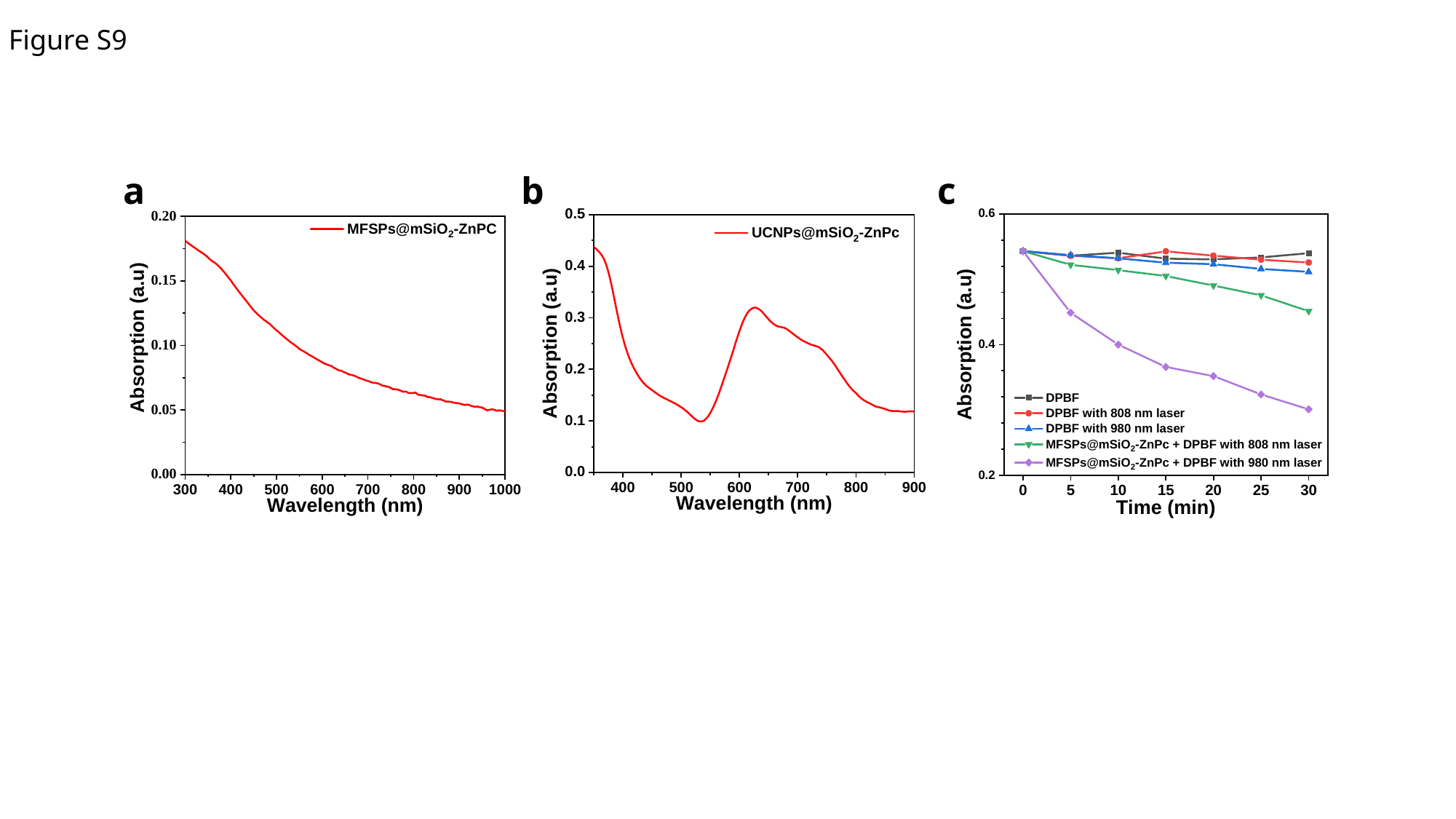

Figure S9
a
b
c
